# Toward Doubly Local Double Hybrid Functionals Using Neural-Network Local Mixing Functions

**DOI:** 10.1021/acs.jctc.5c01952

**Published:** 2026-02-13

**Authors:** Nóra Kovács, Szymon Śmiga, Martin Kaupp, Artur Wodyński

**Affiliations:** † 26524 Technische Universität Berlin, Institut für Chemie, Theoretische Chemie/Quantenchemie, Sekr. C7, Straße des 17. Juni 135, D-10623 Berlin, Germany; ‡ Institute of Physics, Faculty of Physics, Astronomy and Informatics, 317747 Nicolaus Copernicus University in Toruń, ul. Grudziądzka 5, 87-100 Toruń, Poland

## Abstract

The first optimization and evaluation of “doubly local double hybrid” (DLDH) functionals is reported. DLDHs provide a position-dependent mixing not only of exact and semi-local exchange but at the same time also of second-order perturbational (PT2) and semilocal correlation. Both admixtures are governed by local mixing functions (LMFs) in coordinate space. The LMFs have been trained as neural networks, where the correlation LMF is either trained independently or fixed during training to the square of the exchange LMF (DL^2^DH variant). Training and evaluation has been done for different combinations of W4–17 atomization-energy and BH76 barrier test sets with the Slim16 or Slim20 subset of the large GMTKN55 benchmark suite. Comparison is also made to local double hybrids exhibiting constant PT2 admixtures and to local hybrids, trained in the same way, and an application to the argon-benzene dissociation curve is provided. As training-set choices are currently limited by the availability of only an inefficient implementation of the PT2 energy density, this work serves as an initial evaluation of feasibility before implementing more efficient versions. Graphical comparisons of differently trained LMFs for both exchange and correlation provide appreciable insights into the function of DLDHs. Interestingly, we find that the best training runs make the addition of empirical dispersion corrections obsolete for optimum performance for either the Slim16 or Slim20 subsets. This can be traced to very large values of the correlation LMFs in spatial regions where noncovalent interactions originate. This observation indicates possible advantages of position-dependent PT2 admixtures in DLDHs, while the position-dependent exchange admixture offers the potential for subsequent inclusion of strong-correlation effects.

## Graphical abstract

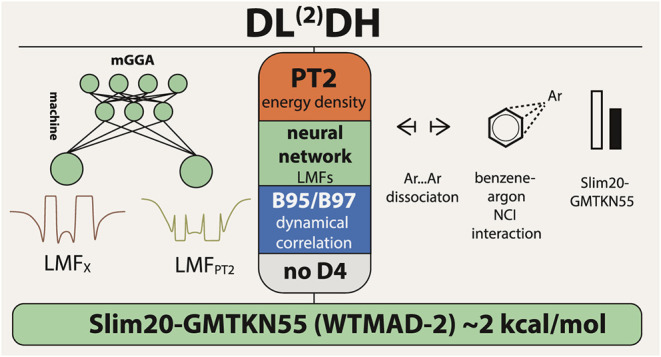

## 1 Introduction

Kohn–Sham density functional theory (KS-DFT) is the central workhorse of modern electronic structure theory, widely used across chemistry, biology, solid-state physics, and materials science.^1–3^ Its success rests on balancing computational efficiency with the systematic improvement of approximate exchange–correlation (XC) functionals. The efforts of our lab in constructing improved density-functional approximations (DFAs) have centered on exploiting the flexibility of the exact-exchange (EXX) energy density.^4,5^ Most recently, this has led to local hybrid and range-separated local hybrid DFAs (LHs and RSLHs) that offer a remarkable escape from the usual “zero-sum game” ^6,7^ between reducing delocalization errors on one side and static-correlation errors on the other side.^8,9^ This has been achieved by including strong-correlation factors into the local mixing functions (LMFs) that determine the real-space position-dependence of the EXX admixture in LHs and RSLHs. Recently, we have also applied machine learning to shape the LMF in LHs.^10^ This has so far culminated in the LH25nP functional,^11^ which combines a strong-correlation factor with a neural-network LMF (“n-LMF”) and achieves outstanding performance for both weakly correlated main-group systems and spin-restricted bond dissociation, as well as for other measures of static correlation.

These developments pertain to the fourth or “hyper-GGA” rung of the usual “Jacob’s ladder” hierarchy^12^ of DFAs. While we expect further substantial improvements on rung 4 in future, we may ask if position-dependent admixtures of exchange and correlation energy densities may also be a way forward on the fifth and highest, fully nonlocal rung of the ladder. Double hybrid functionals (DHs) are the most prominent rung-5 functionals at present.^13–15^ They typically combine a global hybrid (GH), i.e., a constant EXX admixture on the exchange side, with an equally global admixture of semilocal correlation and a second-order perturbation contribution along the lines of Görling-Levy PT2 ^16^ (equivalent to MP2 when using Hartree–Fock orbitals). The latter term brings in nonlocal correlation involving the virtual orbitals and eigenvalues of the system and thereby elevates the DHs to rung 5. DHs are currently the most accurate, widely applied functionals for systems with moderately strong correlations, as exemplified by the popular GMTKN55 ^17^ benchmark suite.^18^ As was recently shown,^19^ present-day DHs still have non-negligible functional-driven errors^20^ and obtain their relatively good performance due to certain effects of error compensation. When combined with empirical dispersion corrections, they can reach weighted mean absolute deviations (WTMAD-2 values) of around 2 kcal/mol for GMTKN55.^15,18,21^ DHs are not successful for systems with strong electron correlations,^22,23^ however, as both regular PT2 correlation and the large global EXX admixture are not well suited for systems with static correlations and/or small band gaps. There have been some efforts to extend DHs to the treatment of such systems,^24–26^ and there are other rung 5 functionals like the so-called σ-functionals that may be applicable to such situations.^27–30^

To what extent could the advantages of position-dependent EXX admixture also be used with rung 5 functionals to obtain further improved DFAs? We recently explored the first range-separated local double hybrid (RSLDH) functionals that combined an RSLH with a (constant) mixing between semilocal and PT2 correlation.^31^ A manual design of the LMF for such LDHs led either to vanishing PT2 contributions or to a loss of spatial variation in the EXX admixture (i.e., the LMF collapsed to a constant).^31^ We therefore used an n-LMF approach (see ref 32 for a recent review), i.e., we trained the LMF as a shallow neural network. This did indeed provide the first LDH functionals, with physically explainable n-LMFs and post-SCF WTMAD-2 values below 2 kcal/mol. Yet the improvement upon the underlying (RS)­DH with constant EXX admixture was only about 0.3 kcal/mol,^31^ which is much less than what can be achieved with (RS)­LHs compared to GHs or range-separated (global) hybrids.

We speculate that the constant admixture of PT2 correlation into the overall correlation functional limits the flexibility of combining PT2 correlation and LH exchange. DHs tend to exhibit very large constant EXX admixtures, and the LMFs of the LDHs of ref 31 also generally showed very large values in most regions of coordinate space. While range separation has been applied earlier to the mixing of DFT and wave function contributions in correlation functionals,^33–38^ to the best of our knowledge, a real-space position-dependent mixing of correlation-energy densities has so far not been evaluated. However, we note that PT2 energy densities have been used in a different type of rung-5 functionals to approximate the initial slope for local interpolations of the adiabatic connection at λ = 0.^39,40^ This corresponds to a somewhat different philosophy than the direct mixing of PT2 and semilocal correlation-energy densities. Here we provide a first extension of DHs and LDHs to a “doubly local double hybrid” (DLDH) framework, where both EXX exchange and PT2 correlation admixtures are done in a position-dependent manner using LMFs for exchange and correlation. We will also construct DLH and LH functionals with LMFs trained in the same way for comparison. Note that the present work is a first attempt using nonoptimized Python code associated with PySCF^41^ to compute exact-exchange and PT2-correlation energy densities. This severely limits the size of molecules we can use for training and evaluation. In this initial work, we therefore aim at evaluating the feasibility and potential of such DLDHs as a prerequisite for deciding on the usefulness of efficient implementations and/or the development and use of lower-rung surrogates^42^ for the PT2 energy density. We will also not attempt to include corrections for strong correlation, which in any case would be incompatible with the unregularized PT2 expressions used.

## 2 Theory

We start with GHs that use a fixed EXX admixture to reduce self-interaction errors (SIE)

1 $$E_{\mathrm{xc}}^{\mathrm{GH}}=aE_{X}^{\mathrm{ex}}+\left(1-a\right)E_{X}^{\mathrm{sl}}+E_{\mathrm{DC}}^{\mathrm{sl}}$$

where *a* is a constant mixing coefficient, *E*_X_^ex^ denotes exact Hartree–Fock exchange, and *E*_X_^sl^ and *E*_DC_^sl^ are the semilocal exchange and correlation contributions, respectively. GHs are clearly unable to approach a solution to the zero-sum game (see Section 1), and this problem also extends to DHs.

LHs instead use a point-wise variation of the EXX energy density, governed by the LMF *a*_X_(**r**), providing the exchange mixing

2 $$E_{\mathrm{xc}}^{\mathrm{LH}}=E_{X}^{\mathrm{ex}}+\int \left(1-a_{X}\left(r\right)\right)\sum_{\sigma }\left(e_{X,\sigma }^{\mathrm{sl}}\left(r\right)-e_{X,\sigma }^{\mathrm{ex}}\left(r\right)\right)dr+E_{c}^{\mathrm{sl}}$$

where *e*_X,σ_^ex^(**r**) and *e*_X,σ_^sl^(**r**) are the exact and the semilocal exchange-energy density, respectively. In the MO basis, the exact-exchange energy density is defined as

3 $$e_{X,\sigma }^{\mathrm{ex}}\left(r\right)=-\frac{1}{2}\sum_{i,j}\phi_{i}^{*}\left(r\right)\phi_{j}\left(r\right)\int \frac{\phi_{i}\left(r^{\prime }\right)\phi_{j}^{*}\left(r^{\prime }\right)}{\left|r-r^{\prime }\right|}dr^{\prime }$$

*e*_X,σ_^ex^(**r**) is defined in the Coulomb gauge, i.e., in terms of the Coulomb potential generated by the exchange hole, consistent with the gauge employed in ref 39.

This local modulation provides an effective way to mitigate the zero-sum game between the reduction of SIE and the simulation of static correlation.^8^ The form used in eq 2 is chosen differently from that of eq 1 to better show that a middle term describing static correlation is added to full EXX (*E*_X_^ex^) and a semilocal dynamical correlation functional (*E*_c_^sl^). As an LH can provide the correct asymptotic energy density but not the correct asymptotic potential at small densities,^43^ extensions have been made to RSLHs featuring full long-range EXX,^44,45^ more recently modified by strong-correlation terms.^46^ RSLHs combine range-separation and real-space EXX variation and can be written as

4 $$E_{\mathrm{xc}}^{\mathrm{RSLH}}=E_{X}^{\mathrm{ex}}+\int \left(1-a_{X}\left(r\right)\right)\sum_{\sigma }\left(e_{X,\sigma }^{\mathrm{sl},\omega }\left(r\right)-e_{X,\sigma }^{\mathrm{ex},\omega }\left(r\right)\right)dr+E_{\mathrm{DC}}^{\mathrm{sl}}$$

where ω is the range-separation parameter governing the partitioning of the exact and semilocal exchange-energy densities into short- and long-range parts in interelectronic-distance space. In strong-correlation corrected RSLHs the middle term is further modified to allow long-range contributions to static correlation to enter (not shown here).^8,9,46^

In an attempt to extend RSLHs to rung 5, we recently^31^ evaluated the first RSLDHs with a constant fraction of PT2 correlation. For consistency with the DLDHs described below, the LDH variant considered here does not use range separation,

5 $$E_{\mathrm{xc}}^{\mathrm{LDH}}=E_{X}^{\mathrm{ex}}+\int \left(1-a_{X}\left(r\right)\right)\sum_{\sigma }\left(e_{X,\sigma }^{\mathrm{sl}}\left(r\right)-e_{X,\sigma }^{\mathrm{ex}}\left(r\right)\right)dr+\left(1-c_{\mathrm{PT}2}\right)E_{\mathrm{DC}}^{\mathrm{sl}}+c_{\mathrm{PT}2}E_{\mathrm{PT}2}$$

where *c*_PT2_ is a global scaling factor for the PT2 energy contributions,

6 $$E_{c}^{\mathrm{PT}2}\approx -\sum_{\mathrm{ijab}}\frac{\left(\mathrm{ia}|\mathrm{jb}\right)\left[2\left(\mathrm{ia}|\mathrm{jb}\right)-\left(\mathrm{aj}|\mathrm{bi}\right)\right]}{\varepsilon_{i}+\varepsilon_{j}-\varepsilon_{a}-\varepsilon_{b}}$$

where indices (*i*, *j*) and (*a*, *b*) refer to occupied and virtual MOs, respectively. Note that in ref 31, additionally spin-scaled PT2 contributions were used. Here we show just a single PT2 coefficient, as in the present work we will not use spin-scaling in DLDHs for simplicity, and therefore decided not to use it either for the optimization of LDHs.

Given the limited flexibility of EXX mixing in the presence of spatially constant PT2 contributions^31^ (see Section 1), we propose a DLDH framework in this work, introducing a correlation LMF *a*_PT2_(**r**) to modulate the PT2 energy density in coordinate space. This requires revisiting the gauge problem of energy densities in local hybrids (LHs). Exchange^5,47,48^ and correlation^39^ energy densities are not uniquely defined. They can be modified by adding any function that integrates to zero. While such modifications do not affect globally integrated energies, they do alter local energy densities. Upon mixing with a position-dependent LMF, the final energy becomes gauge-dependent. To address this for exchange, previous studies introduced an optimized calibration function (CF) integrating to zero to minimize artifacts from this ambiguity.^4,47,48^ Recently, we have shown that neural-network-based LMFs can implicitly suppress unphysical contributions from problematic regions, rendering explicit CFs unnecessary.^10,49^ In this work, we will assume that this also holds for *a*_PT2_(**r**), and we will thus not introduce CFs at all. Then a DLDH can be formulated as

7 $$E_{\mathrm{xc}}^{\mathrm{DLDH}}=E_{X}^{\mathrm{ex}}+\int \left(1-a_{X}\left(r\right)\right)\sum_{\sigma }\left(e_{X,\sigma }^{\mathrm{sl}}\left(r\right)-e_{X,\sigma }^{\mathrm{ex}}\left(r\right)\right)dr+\int \left[\left(1-a_{\mathrm{PT}2}\left(r\right)\right)e_{\mathrm{DC}}^{\mathrm{sl}}\left(r\right)+a_{\mathrm{PT}2}\left(r\right)e_{c}^{\mathrm{PT}2}\left(r\right)\right]dr$$

where *e*_c_^PT2^(**r**) is defined as

8 $$e_{c}^{\mathrm{PT}2}\left(r\right)\approx -\sum_{\mathrm{ijab}}\frac{{\left(\mathrm{ia}|\mathrm{jb}\right)}^{r}\left[2\left(\mathrm{ia}|\mathrm{jb}\right)-\left(\mathrm{aj}|\mathrm{bi}\right)\right]}{\varepsilon_{i}+\varepsilon_{j}-\varepsilon_{a}-\varepsilon_{b}}$$

9 $${\left(\mathrm{ia}|\mathrm{jb}\right)}^{r}=\phi_{a}^{*}\left(r\right)\phi_{i}\left(r\right)\int \frac{\phi_{j}\left(r^{\prime }\right)\phi_{b}^{*}\left(r^{\prime }\right)}{\left|r-r^{\prime }\right|}dr^{\prime }$$

As in ref 39, we employ here the Coulomb gauge in our definition of the PT2 energy density, more precisely that of the electrostatic potential of the exchange-correlation hole. We also note that eq 8 uses an approximation to the exact Görling-Levy *e*_c_^PT2^(**r**) expression, as no single-excitation contributions are included. These are known to be small.^39,50^ That is, we use just the MP2-like operator evaluated on Kohn–Sham orbitals, as is common for DHs (see also eq 6). Consequently, the grid-integrated *e*_c_^PT2^(**r**) energy provides an approximation to *E*_PT2_, with an accuracy that depends on the quality and resolution of the numerical grid. We note in passing that this standard MP2-like expression will diverge with closing band gap, e.g., for the spin-restricted dissociation of covalent bonds. Such situations are clearly outside the scope of the present work, as we also do not attempt to include any correction terms for static correlations.^8,9,11^ It is clear that an extension to this area would also require us to use modified PT2 expressions, either regularized ones^23^ or correlation functionals based on the adiabatic-connection fluctuation-dissipation theorem. Also, for simplicity we deliberately base the present approach on full-range exchange-energy densities. That is, we do not employ a range-separated local hybrid (RSLH) form, but rather start from a conventional LH expression.

In eq 7 the two LMFs *a*_X_(**r**) and *a*_PT2_(**r**) are independent quantities. For DHs, arguments have been put forward that a nonempirical framework should provide a PT2 coefficient that is the square of the EXX coefficient in the exchange part.^51^ We will therefore also explore a coupling of the two LMFs during training [*a*_PT2_(**r**) = *a*_X_(**r**)^2^] and call the resulting functionals the DL^2^DH variants. Additionally, we explored a softer coupling between the two LMFs during training. Rather than enforcing the strict quadratic relation as in the DL^2^DH model, we introduced a penalty term that discourages deviations from this relation

10 $$L_{\mathrm{penalty}}=c\left\langle {\left[a_{\mathrm{PT}2}-{\left(a_{X}\right)}^{2}\right]}^{2}\right\rangle$$

where prefactor *c* controls the strength of coupling between the exchange and correlation LMFs. This variant is referred to as the “approximately coupled” (DL^*ac*^DH) model in the following. Small values of *c* correspond to a weak coupling, allowing the two LMFs to vary more or less independently, while larger values enforce the quadratic relationship more strictly. We tested *c* = 0.05, 0.5, 2, and 5, representing progressively stronger coupling. The penalty function was added to the total training loss, enabling a continuous interpolation between the fully decoupled DLDH model and the strictly constrained DL^2^DH variant.

### 2.1 Setup of the Neural-Network LMFs

Similar to our previous works on n-LMFs for DLHs,^31^ and LHs,^10,11^ the LMF is constructed as a multilayer perceptron (MLP). Three hidden layers of 128 GeLU-activated^52^ neurons each and a sigmoid-activated output layer are used, ensuring that the outputs remain within the physically meaningful interval [0, 1] (as strong-correlation effects are not addressed in this work, we neither require nor permit negative values of the LMF^11^). In compact form, an *L*-layer MLP can be written as

11 $$a^{n}\left(r\right)=\phi_{\mathrm{out}}\left(W_{L+1}\phi_{L}\left(\cdot \cdot \cdot \phi_{1}\left(W_{1}x\left(r\right)+b_{1}\right)\cdot \cdot \cdot \right)+b_{L+1}\right)$$

where **W**_*i*_ and **b**_*i*_ denote the trainable weight matrices and bias vectors for layer *i*, respectively, and each ϕ_*i*_ is a GeLU activation function. In eq 11, the network output *a* ^*n*^(**r**) may either be a scalar, interpreted as *a*_X_(**r**) with *a*_PT2_(**r**) computed as its square (DL^2^DH case), or a two-element vector directly providing both *a*_X_(**r**) and *a*_PT2_(**r**) (DLDH case). The simpler LDH and LH models employ only the scalar version with the output corresponding to *a*_X_(**r**).

The input features given to the MLP are meta-GGA quantities. They comprise the spin resolved electron densities (ρ_α_, ρ_β_), the squared norms of their gradients (∇ρ_α_·∇ρ_α_, ∇ρ_β_·∇ρ_α_, ∇ρ_β_·∇ρ_β_), and kinetic-energy densities (τ_α_, τ_β_). To ensure numerical stability and accelerate training convergence, all input features are preprocessed using a logarithmic “squashing” transformation prior to being passed to the neural network.^10^ The network is evaluated twice to preserve spin symmetry, once with the (α, β) ordering and once with order (β, α), and the outputs are averaged to produce the final n-LMF(s). Further details are provided in Supporting Information.

### 2.2 Parametrization of the Remaining Parts of the Exchange Correlation Functional

In the present work, all calculations were performed within an xDLDH-type framework, in which the Kohn–Sham orbitals are generated from a fixed GH (see Section 3 below), and the DFT and PT2 contributions to the final energy are evaluated in a post-SCF manner with orbitals obtained from the GH. This approach has been chosen primarily for its simplicity and limited computational training cost. A gDLDH formulation with self-consistent treatment of all contributions except for the post-SCF PT2 term is also conceivable. However, a gDLDH implementation within the current PySCF infrastructure would require substantial code modifications and is therefore beyond the scope of the present initial work.

The final functional combines EXX and PBE^53^ exchange and EXX with either a semilocal B95c^54^ or B97c^55^ dynamical correlation functional, augmented by the PT2 term. Notably, in this initial study, we did not attempt to reoptimize the internal parameters of the semilocal correlation functionals, apart from the modulation introduced via *a*_PT2_. The parameters for B95c were adopted from the LH20t functional,^4^ while those for B97c were taken from LH24n^10^ (see Table S1 in Supporting Information). That is, optimization in the present work is limited to the LMFs, and to the added D4 dispersion^56^ corrections (see below).

### 2.3 Training Protocol and Data Sets

Figure 1 provides a schematic overview of the training and evaluation workflow. The primary data sets were the W4–17 set of atomization energies^57^ and the BH76 set of barrier heights,^58,59^ which were also employed in earlier n-LMF developments.^10^ To extend chemical diversity and include noncovalent interactions (NCIs), additional data were sought from the GMTKN55 benchmark suite.^17^ Given the current limitations in system sizes that can be afforded during training and evaluation, we decided to focus on the recently proposed Slim20 and Slim16 subsets^60^ of GMTKN55, limited to molecules of at most 20 or 16 atoms, respectively, while retaining good data diversity. The loss function for LMF training was defined as a weighted mean absolute deviation, where the BH76 and W4–17 sets were assigned weights of 1.0 and 0.5, respectively, and the Slim20 or Slim16 subset were weighted to reproduce the WTMAD-2 value for the respective Slim-GMTKN55 subset. The overall loss function is given by

12 $$L_{\mathrm{total}}=1.0L_{\mathrm{BH}76}+0.5L_{W4‐17}+\sum_{r\in \mathrm{Slim}16/20}w_{r}^{\mathrm{WTMAD}‐2}L_{r}$$

**Figure 1:**
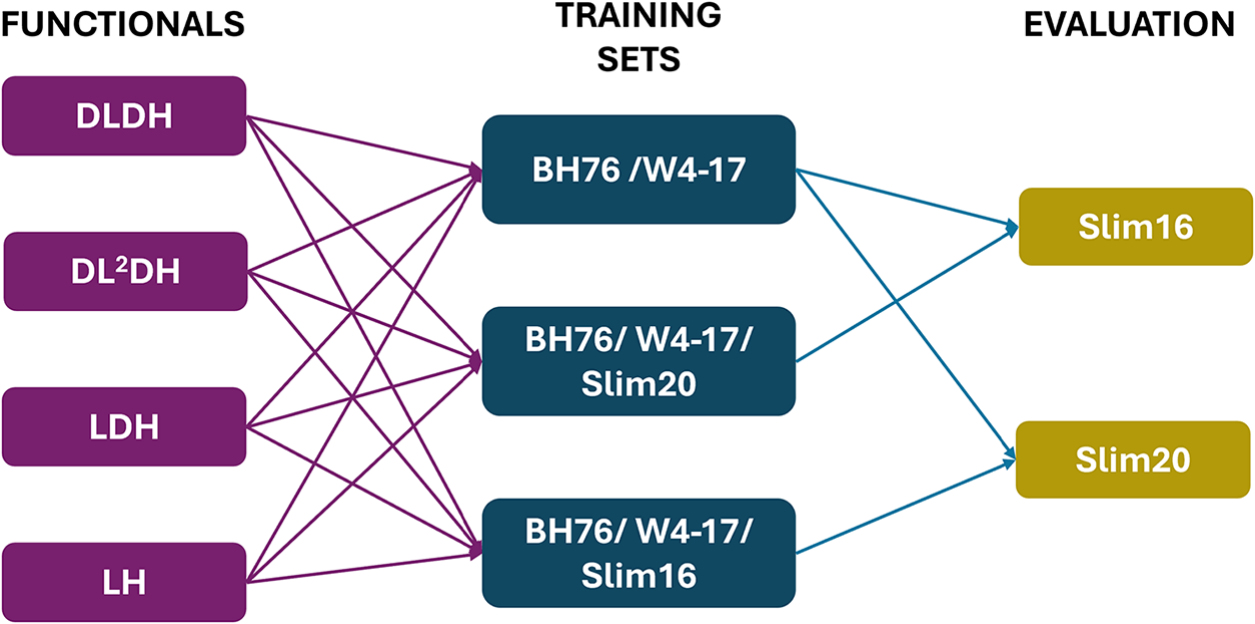
Overview of the training and evaluation workflow, including types of functionals and composition of test and evaluation sets.

Dispersion interactions play a significant role in certain parts of the Slim20 and Slim16 subsets. Therefore, two levels of training protocols were employed. First, the functionals were trained on the BH76 and W4–17 data sets and separately on the extended BH76/W4–17/Slim-GMTKN55 (including Slim20 or Slim16 subsets) combinations, both without any dispersion corrections. In a second step, the n-LMF was fixed and D4 dispersion corrections^56^ were incorporated by optimizing the D4 parameters (*a*_1_, *a*_2_, *s*_8_, and *s*_9_) against reduced subsets with systems up to 20 atoms of the S22 ^61^ and S66 ^62^ sets, to ensure computational feasibility for the PT2 energy-density evaluations. The resulting optimized D4 parameters were then fixed and used during repeated n-LMF training.

Models trained on different data sets, BH76/W4–17 alone or combined with Slim20 or Slim16, were cross-evaluated on Slim16 and Slim20, respectively, to assess transferability (see Figure 1). BH76/W4–17-trained models were evaluated both on Slim20 and on Slim16 for completeness.

## 3 Computational Details

Molecular orbitals were generated with the PySCF 2.6.2 ^41^ package using PBE-based GHs, namely PBE0 (25% EXX), PBE60 (60% EXX), and PBE80 (80% EXX), in combination with def2-QZVP basis sets,^63^ a numerical grid of level 1, and the PySCF default SCF convergence threshold of 10^–9^ Hartree. For all elements with atomic number *Z* > 36 (i.e., beyond krypton), an effective core potential (ECP)^64^ was employed. From the PBE-based GH orbitals, spin-resolved meta-GGA input features were computed using an in-house Python program interfaced with PySCF, along with the EXX and PT2 energy densities, the latter implemented in a relatively memory-efficient batched UPT2 approach without the frozen-core approximation

The network was implemented in Python using the TensorFlow library.^65^ The training was carried out for 2000 epochs. In addition to the default architecture with three hidden layers of 128 neurons, a smaller network variant comprising two hidden layers of 64 neurons each was also tested for the DLDH and DL^2^DH functionals. A detailed performance comparison is provided in Tables S5 and S6 in Supporting Information.

For computation of the argon–benzene dissociation curves, larger basis sets (def2-QZVPPD) were employed. The curves represent the distance between the Ar atom and the center of the benzene ring in C_6v_ symmetry. Two different integration grids were evaluated: the default level 1 grid of PySCF, used to maintain consistency with the neural-network training, and a larger (99,590) grid, which enables direct comparison with the results reported in ref 66.

All Python scripts used for orbital generation, neural-network training, post-SCF evaluations of the n-LMFs (including the PBE exchange and B95c/B97c correlation implementations), as well as optimization of dispersion parameters, are publicly available on GitHub.^67^

## 4 Results

### 4.1 Dependence of Shape and Quality of the LMF on the Chosen Input Orbitals

Since both the training and evaluation of the new functionals are performed in a post-SCF manner using orbitals obtained from PBE-based GHs, it is essential to first assess the dependence of the learned LMFs on the choice of input orbitals. As was shown in ref 19, the quality of second-order correlation contributions depends strongly on the reference orbitals used. Figure 2 illustrates the resulting *a*_X_(**r**) for the CS molecule using DLDH functionals combined with B95c correlation, based on input orbitals from PBE0, PBE60, and PBE80, corresponding to 25%, 60%, and 80% EXX admixture, respectively.

**Figure 2:**
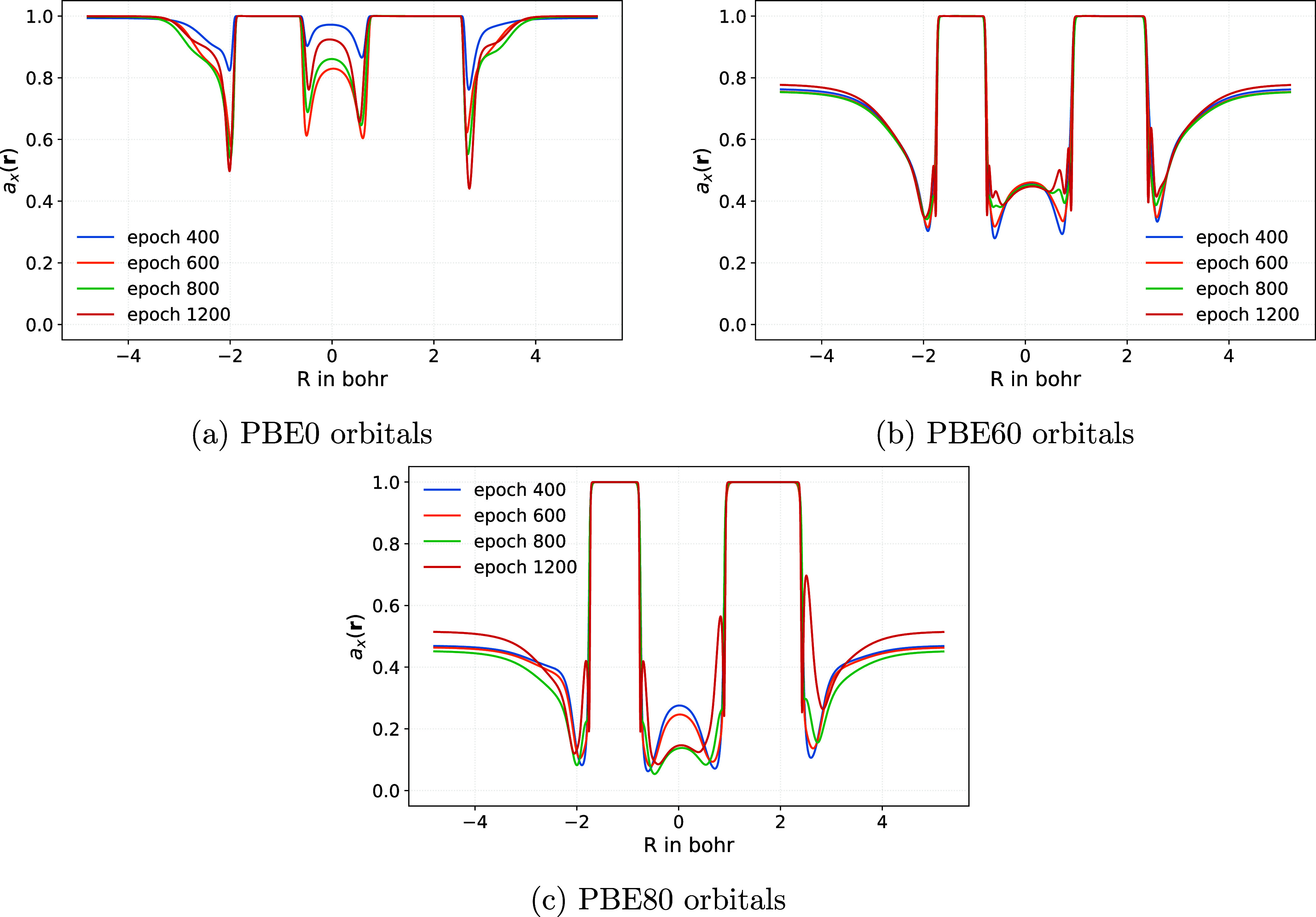
Comparison of LMFs for exchange in the DLDH functional with B95c correlation for the CS molecule, trained on the W4–17 and BH76 data sets. Results are shown for (a) PBE0, (b) PBE60, and (c) PBE80 orbitals at training epochs 400, 600, 800, and 1200.

A clear and systematic trend is observed: increasing the EXX admixture in the underlying GH functional leads to lower average values of the learned *a*_X_(**r**). This behavior suggests that the neural-network-based LMF compensates for density-driven errors^20^ present in orbitals with lower EXX content by assigning a larger local EXX admixture during post-SCF energy evaluation. As the input orbitals become less affected by SIE with increasing EXX contributions, the trained LMF correspondingly applies a smaller correction.

Table S4 in Supporting Information shows that LMFs based on training with PBE60 orbitals generally produce the smallest mean absolute errors (MAEs) for W4–17 atomization energies and BH76 barriers among the trained rung 5 functionals. These findings suggest that PBE60 orbitals provide a favorable balance between reduced density-driven errors and sufficient flexibility in the trained LMF. This choice is further supported by the observation that most established DHs employ EXX admixtures in the 50–80% range. We will therefore focus in the following on calculations with PBE60 orbitals.

### 4.2 Comparing Performance of DLDH, DL^2^DH, LDH, and LH Functionals Obtained with Different Training Protocols

Figure 3 presents WTMAD-2 values for the Slim16 and Slim20 evaluation subsets, comparing various functionals trained under different protocols. We include DLDH, DL^2^DH, LDH, and LH models combined with either B95c or B97c correlation, with and without D4 dispersion corrections. Full numerical results are provided in Tables S11 and S12 in Supporting Information.

**Figure 3:**
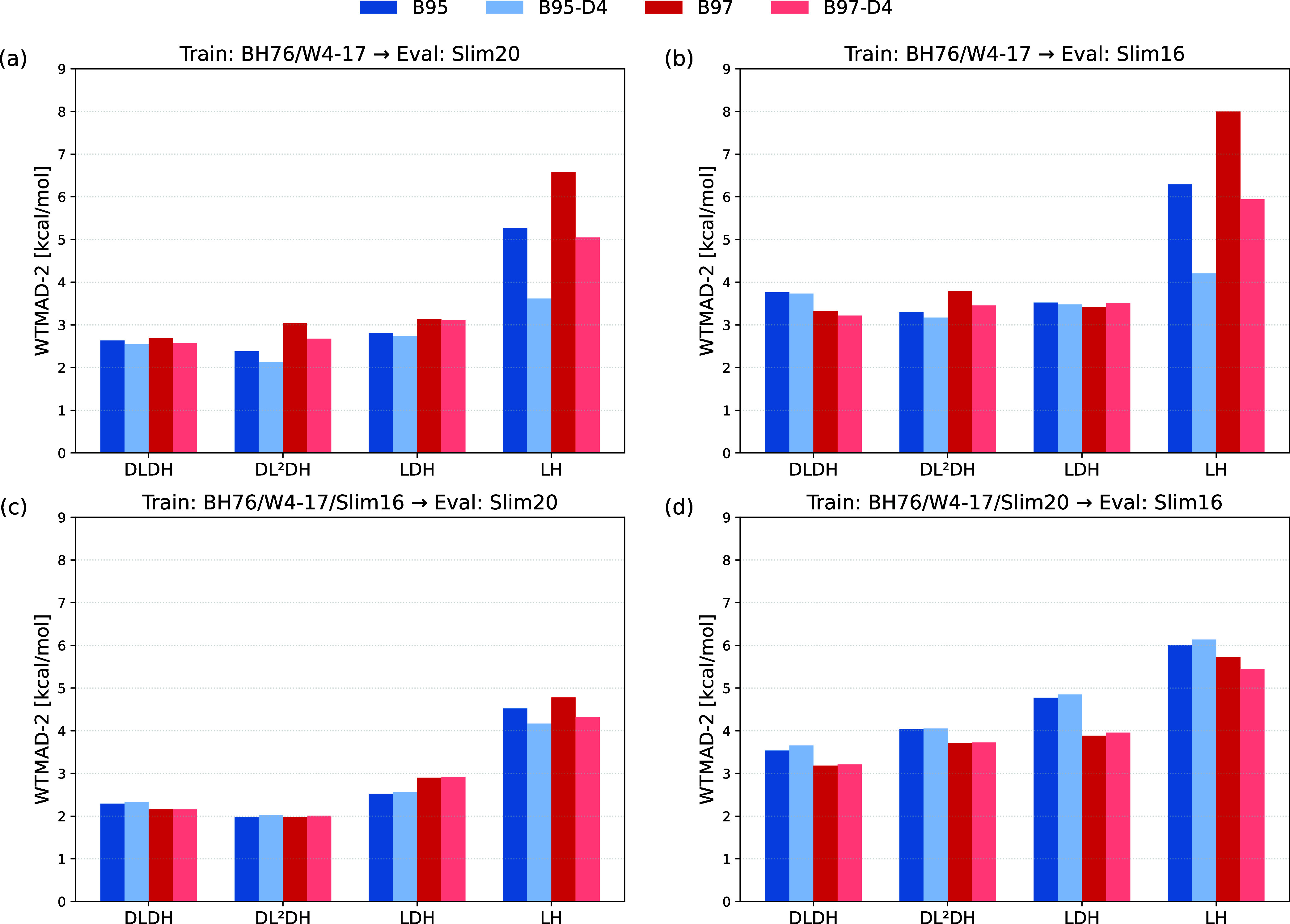
WTMAD-2 (kcal/mol) for B95c- and B97c-based correlation variants of DLDH, DL^2^DH, LDH, and LH functionals. Results are shown for four training → evaluation combinations: (a) BH76/W4–17 → Slim20, (b) BH76/W4–17 → Slim16, (c) BH76/W4–17/Slim16 → Slim20, and (d) BH76/W4–17/Slim20 → Slim16. Bars compare performance without dispersion correction (dark) and with D4 dispersion correction (light).

Panels (a) and (b) show results for LMFs trained solely on the BH76 and W4–17 sets and evaluated using the Slim20 and Slim16 subsets, respectively. For functionals that include a PT2 term (DLDH, DL^2^DH, LDH), the D4 correction has only a minor effect on WTMAD-2, typically changing the values by no more than a few tenths of a kcal/mol. In contrast, the impact is more significant for LH models, which lack a PT2 component and thus rely more heavily on empirical dispersion.

Notably, when the LMF is trained on a broader data set, including the Slim16 or Slim20 subsets in addition to the BH76 and W4–17 sets, the impact of D4 corrections on WTMAD-2 is substantially reduced (see bottom panels c and d in Figure 3). For LH models, the contribution from D4 becomes modest, and for DLDH, DL^2^DH, and LDH functionals, it is negligible or even slightly detrimental (see Table S10 in Supporting Information). This behavior likely reflects the fact that once well-trained LMFs are in place for models that already include a perturbative PT2 contribution, dispersion interactions are effectively captured by the latter. Adding an additional empirical dispersion correction in such cases may lead to mild overcounting or may disrupt delicate error compensation mechanisms already encoded in the LMF and PT2 components. Subsequent discussions of the performance of DLDH and DL^2^DH functionals will therefore focus on results obtained without D4 corrections.

When trained only on BH76 and W4–17 data, the advantage of DLDH and DL^2^DH over LDH functionals in terms of WTMAD-2 is relatively modest (cf. panel a of Figure 3): the DLDH reaches approximately 2.69 kcal/mol for Slim20, compared to 3.14 kcal/mol for the LDH (B97c variant). However, when the training set is extended to include the Slim20 or Slim16 subsets, the performance gap widens noticeably (see, e.g., panel c): including Slim16 in training, the Slim20 WTMAD-2 values decrease to 2.16 kcal/mol for the DLDH and to 1.97 kcal/mol for the DL^2^DH, while the LDH remains at 2.90 kcal/mol (again using B97c). As discussed below, these results place the DLDH and DL^2^DH functionals among the best-performing functionals in comparison to existing DH benchmarks. A position-dependent PT2 admixture appears to offer clear advantages over a constant one, particularly when the training of *a*_PT2_(**r**) includes substantial noncovalent interaction (NCI) data, such as contained in the Slim16 or Slim20 subsets. We further investigate this trend through LMF analyses below.

Evaluations on the Slim16 subset typically yield higher WTMAD-2 values than for the Slim20 counterpart. Overall, the protocol involving training on BH76/W4–17/Slim16 and evaluation on Slim20 (see panel c in Figure 3) emerges as a robust strategy for capturing NCI effects. A full validation on the complete GMTKN55 benchmark is currently not feasible due to various computational constraints (see Section 2). It seems possible, however, that the benefits of position-dependent PT2 admixture could be even more pronounced for larger systems exhibiting significant dispersion interactions.

Across almost all evaluation sets, the DL^2^DH and DLDH functionals exhibit very similar performance. This suggests that a significant portion of the advantages of spatially resolved PT2 scaling can be recovered from simple functional relationships to the position dependence of EXX admixture. In contrast, LDH models, which have position-dependent EXX admixture but lack any spatial dependence in the PT2 contribution, cannot exploit this relationship.

A softer “approximately coupled” relation between *a*_PT2_(**r**) and *a*_X_^2^(**r**), implemented via the penalty term in the training loss (see Section 2), does not yield any notable improvement over either DLDH models with independently learned LMFs or the strictly constrained DL^2^DH variants. As shown in Table S12, only minor advantages appear when the training is limited to the smaller BH76/W4–17 data set, which vanish once larger and more diverse data such as Slim16 or Slim20 are included. This suggests that while the coupling modifies the local behavior of the mixing functions, it does not yet translate into a clear advantage at the integrated energy level. The approach nonetheless provides a flexible framework for exploring physically motivated correlations between exchange and correlation LMFs, which may become more relevant with richer or more diverse training data in future developments.

Figure 4 compares the performance of the new DLDH and DL^2^DH functionals (trained on BH76/W4–17/Slim16, B97c variant) for Slim20-GMTKN55 with that of selected literature functionals. The DLDH functionals achieve WTMAD-2 values approximately 0.4 kcal/mol lower than that of the DSD-BLYP-D3 functional (cf. ref 60). Notably, our previously reported range-separated global double hybrid ωDH25-D4, which achieved a WTMAD-2 value of 2.13 kcal/mol for full GMTKN55, performs worse on Slim20 (WTMAD-2 value of 2.77 kcal/mol). This suggests that, for some functionals, the Slim20 subset may not fully reflect the behavior observed across the complete GMTKN55 database, likely due to its lack of larger molecules with more pronounced dispersion interactions. Nevertheless, the present DLDH functionals already perform remarkably well on the Slim20 subset, even though they include neither explicit dispersion corrections nor spin-scaling in the PT2 term. We note that the Slim16 and Slim20 sets have a very small overlap of nine reactions. We evaluated the effect of this overlap on the final WTMAD-2 values by recomputing them after excluding the common reactions. This led to minimal changes, typically below 0.1 kcal/mol. For simplicity we will thus continue using the full Slim16 and Slim20 sets. We also summarize the MAEs of the individual loss components evaluated at epoch 800 during training, for all four functional classes (DLDH, DL^2^DH, LDH, and LH), in Tables S7–S9 in Supporting Information.

**Figure 4:**
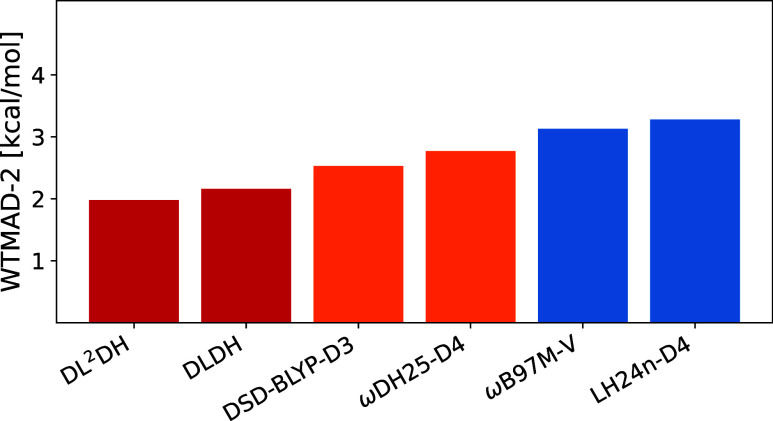
Comparison of Slim20 WTMAD-2 values in kcal/mol for the new DLDH functionals (BH76/W4–17/Slim16 training using B97c) in red with top-performing literature DH (orange), and RSH/LH (blue) functionals.

### 4.3 Further Assessment

An additional validation set we have looked at is the MB08–165 “mindless benchmarking” set,^68^ which contains small molecules with unusual bonding patterns and compositions and thus provides a stringent stress test for generalization. Table S7 in Supporting Information summarizes the resulting MAEs. Depending on the data used for training, DLDHs bracket closely the ≈2.6 kcal/mol MAE of the CCSD­(T)/cc-pVQZ reference level reported in the original MB08–165 study, clearly below the ≈5.6 kcal/mol of the corresponding CCSD calculations or the 4.1 kcal/mol MAE of the DH B2-PLYP-D.^68^ The MAEs of the DL^2^DHs range only a little higher, 2.6–3.8 kcal/mol, depending on training, The DLHs give more notably larger MAEs of 3.3–5.2 kcal/mol, and the LHs perform clearly worse here (7.2–9.8 kcal/mol). We found previously that n-LMF-based LHs like LH24n^69^ and LH25nP^11^ left substantial room for improvement for the related MB16–43 subset of GMTKN55.^17^ This also showcases the advantages of the position-dependent admixture of the PT2 energy density for such less common bonding situations.

To probe if the excellent performance for the BH76 barrier test set might involve error cancellation between transition states and reactants/products, we also computed the related reaction energies of the BH76RC set, which were not included in training. Table S8 reports the corresponding MAEs. Both DLDH and DL^2^DH models provide MAEs below 1 kcal/mol when either Slim16 or Slim20 has been included in the training data. Under these circumstances, the LDHs give around 1.5 kcal/mol, and the LHs provide still slightly higher values. The results for the better-performing DLDH and DL^2^DH models are very similar to those of a recent test of top-performing double hybrid functionals.^15^

### 4.4 Influence of Training Data on LMF Shape

As the LMFs are the key components that modulate exchange and correlation in the functionals studied here, we now proceed to try to better understand how trained LMF shapes in different systems may connect to the overall performance of a given functional. Such graphical analyses support explainability. We focus on LMFs trained with B97c semilocal correlation, further results with B95c and other comparisons are provided in Supporting Information (Figures S1–S6), and we show results after 800 epochs. We begin with the CS molecule (Figure 5) as a representative example of a covalently bound system. The left panels show *a*_X_(**r**), and the right panels show *a*_PT2_(**r**), with DLDH results in the top row and DL^2^DH results in the bottom row. The colored lines correspond to different training protocols: blue for BH76/W4–17 only, green for BH76/W4–17/Slim16, and orange for BH76/W4–17/Slim20 training.

**Figure 5:**
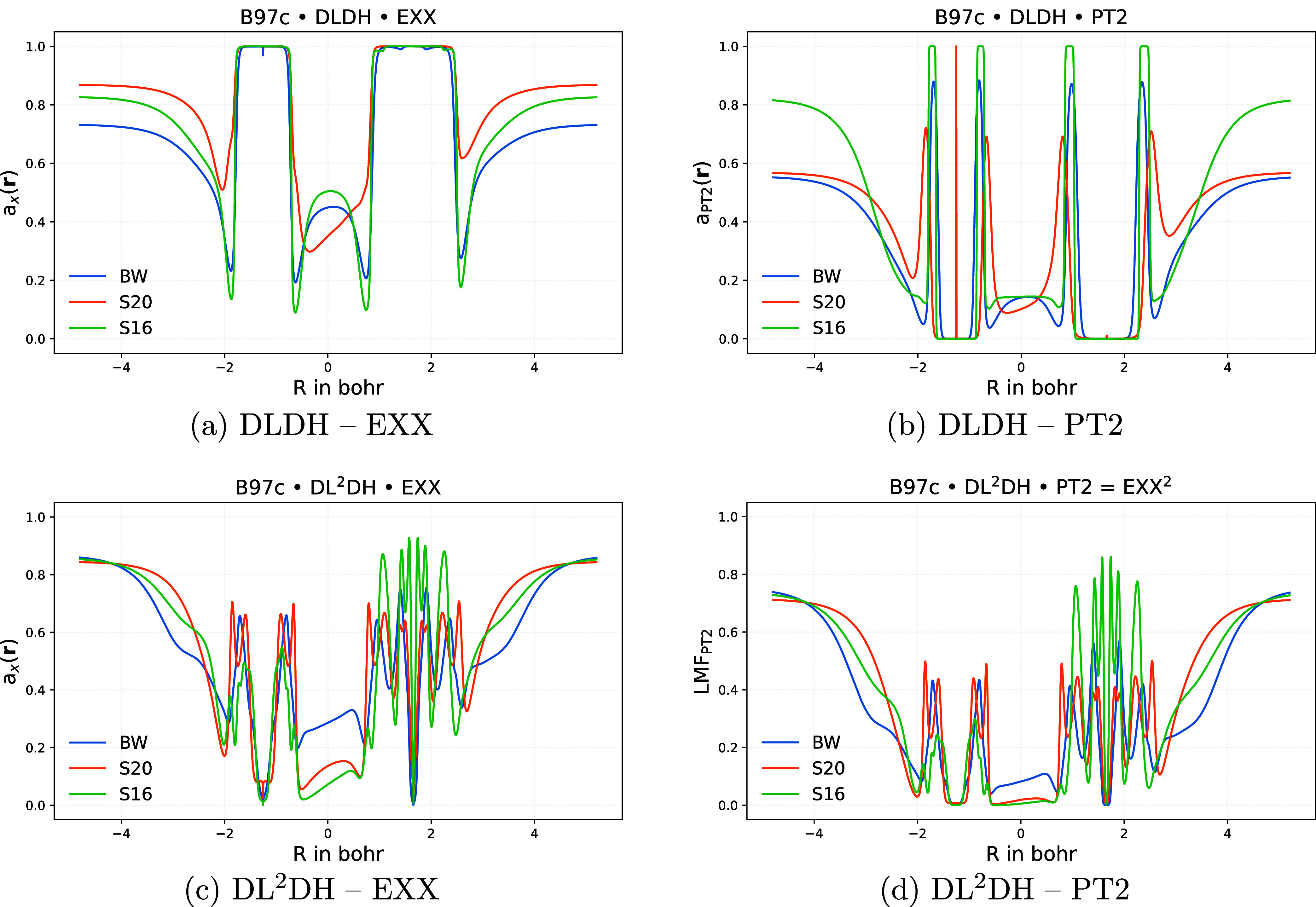
Plots of *a*_X_(**r**) (a, c) and *a*_PT2_(**r**) (b, d) for the CS molecule along the bond axis at epoch 800 for DLDH (a, b) and DL^2^DH functionals (c, d) trained with B97c using different training sets. Abbreviations: BW = BH76/W4–17 training, S16 = BH76/W4–17/Slim16 training, S20 = BH76/W4–17/Slim20 training.

We should start by noting that the core regions of the LMFs are not represented in the training data. This partially explains the pronounced differences observed between DLDH and DL^2^DH in the near-nuclear regions. The DLDH exhibits almost 100% EXX admixture and close to 0% PT2 admixture near the nuclei. In contrast, due to the intrinsic coupling between both LMFs for the DL^2^DHs, their core region shows more internal structure and variability. This could pose a potential drawback of the DL^2^DH protocol if we were to add an optimized core contribution: from a physical perspective *a*_X_(**r**) should approach substantial EXX admixtures near the nuclei that grow with nuclear charge (respecting the high-density coordinate scaling limit^70^) to reflect the dominance of exchange over correlation in the high-density limit,^5^ while the PT2 contribution should simultaneously vanish in this region for the same reason. Such core corrections may, of course, be added on top of suitably designed n-LMFs,^70^ so that the observed differences might only emerge at a later stage of the construction of the LMF.

In the bonding region, the training data have a stronger influence on the shape of both LMFs in the DLDH model than in the DL^2^DHs. This indicates that the coupling between the two LMFs in the latter type of functionals reduces their sensitivity to the training data. Nevertheless, the magnitude of *a*_X_(**r**) within the bond still varies noticeably even for the DL^2^DH model, with the inclusion of either the Slim16 or Slim20 subsets in training leading to overall lower EXX admixture in this region.

Interestingly, the asymptotic values of the LMFs far from the nuclei are also significantly stabilized by the coupling in the DL^2^DH model. In contrast, the DLDH model shows stronger variability depending on the training set: training on BH76/W4–17 alone yields the lowest asymptotic value for exchange, inclusion of the Slim20 subset into training gives the highest exchange asymptote, while inclusion of the Slim16 subset in training leads to the highest PT2 asymptote. As in the near-nuclear region, the training data are not expected to strongly constrain the asymptotic behavior of the EXX admixture. However, an increased PT2 admixture in low-density regions may reflect an improved treatment of dispersion interactions (see below). The coupling of the LMFs in the DL^2^DH model may therefore help enforce more consistent asymptotic behavior in both exchange and correlation components.

Figure S7 in Supporting Information compares the local mixing functions of the approximately coupled DL^*ac*^DH variants to the reference DLDH and DL^2^DH forms for the CS molecule. With increasing coupling strength (larger penalty parameter *c*), the PT2 and EXX LMFs gradually approach the characteristic shapes of the DL^2^DH model, whereas smaller *c* values yield LMFs that remain closer to the uncoupled DLDH form. This systematic trend confirms that the penalty effectively interpolates between the two limiting cases. The increased coupling also introduces fine structure in the core region, reflecting the growing interdependence of the EXX and PT2 channels, yet the relation cannot simultaneously enforce *a*_X_ ≈ 1 in the core and *a*_PT2_ ≈ 0 because of the intrinsic quadratic constraint.

Figure 6 provides in addition plots of *a*_X_(**r**) for the simpler LDH and LH models for the CS molecule. Consistent with earlier observations,^31^ the LDH requires large fractions of exact exchange not only in the input orbitals but also in the LMF in most regions of space (panel a of Figure 6). The EXX admixture in the bonding region appears to be highly sensitive to the inclusion of Slim16 or Slim20 data in training: models trained only on BH76/W4–17 tend to yield significantly larger EXX admixtures in the bond. This is not surprising and aligns with our observations for earlier LDH models^31^ trained exclusively on BH76 and W4–17. We also found recently for the n-LMF of the LH25nP local hybrid,^11^ that the inclusion of more diverse molecules significantly improves the resolution and reliability of the LMF in the bonding region.

**Figure 6:**
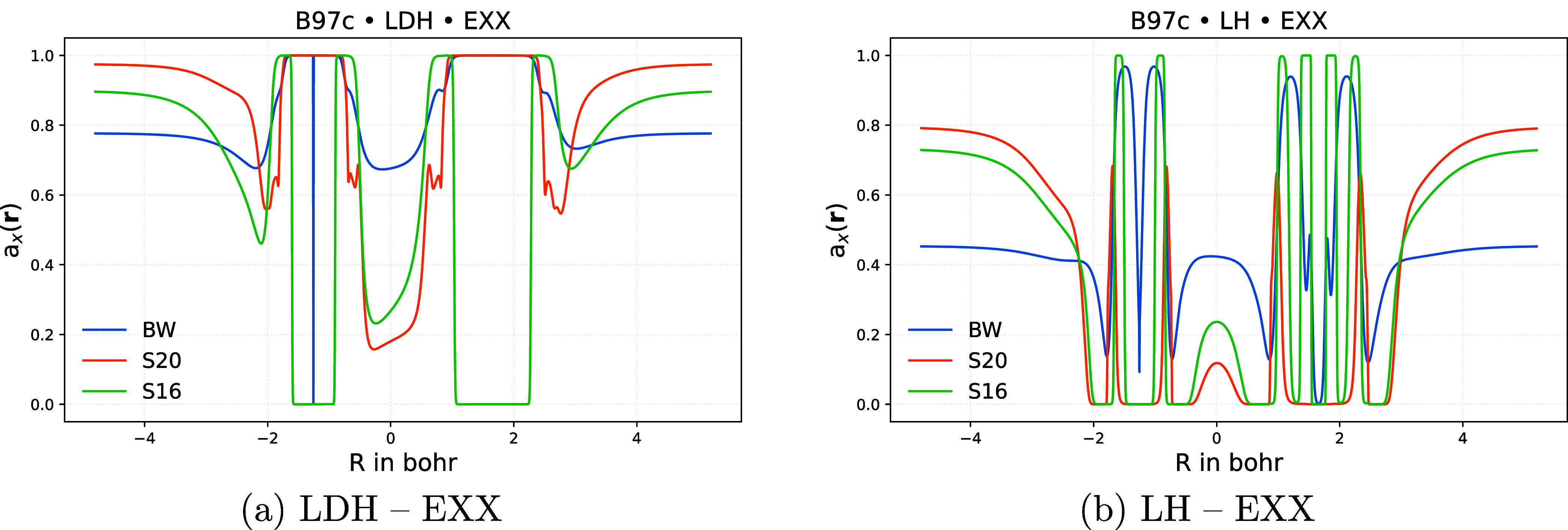
Plots of *a*_X_(**r**) for the CS molecule along the bond axis at epoch 800 for the LDH (a) and LH (b) functionals with B97c correlation using different training sets. Abbreviations: BW = BH76/W4–17 training, S16 = BH76/W4–17/Slim16 training, S20 = BH76/W4–17/Slim20 training.

The LMF of the LH functional is likewise sensitive to the training protocol, with the BH76/W4–17-only model again deviating from training including Slim16 or Slim20 data. Overall, the EXX admixture in the LHs is lower than in the (D)­LDH models, consistent with general trends observed between GHs and LHs, as well as with earlier findings.^31^

Artifacts arising from the gauge problem in *a*_X_(**r**) are known^5^ to manifest mostly in NCIs. And dispersion interactions, for which appropriately large values of *a*_PT2_(**r**) in the interatomic region are desirable, are also most pronounced for NCIs. To analyze these aspects, Figure 7 shows LMFs for DLDH and DL^2^DH functionals for the Ar_2_ dimer at an internuclear distance of 7.5 bohr.

**Figure 7:**
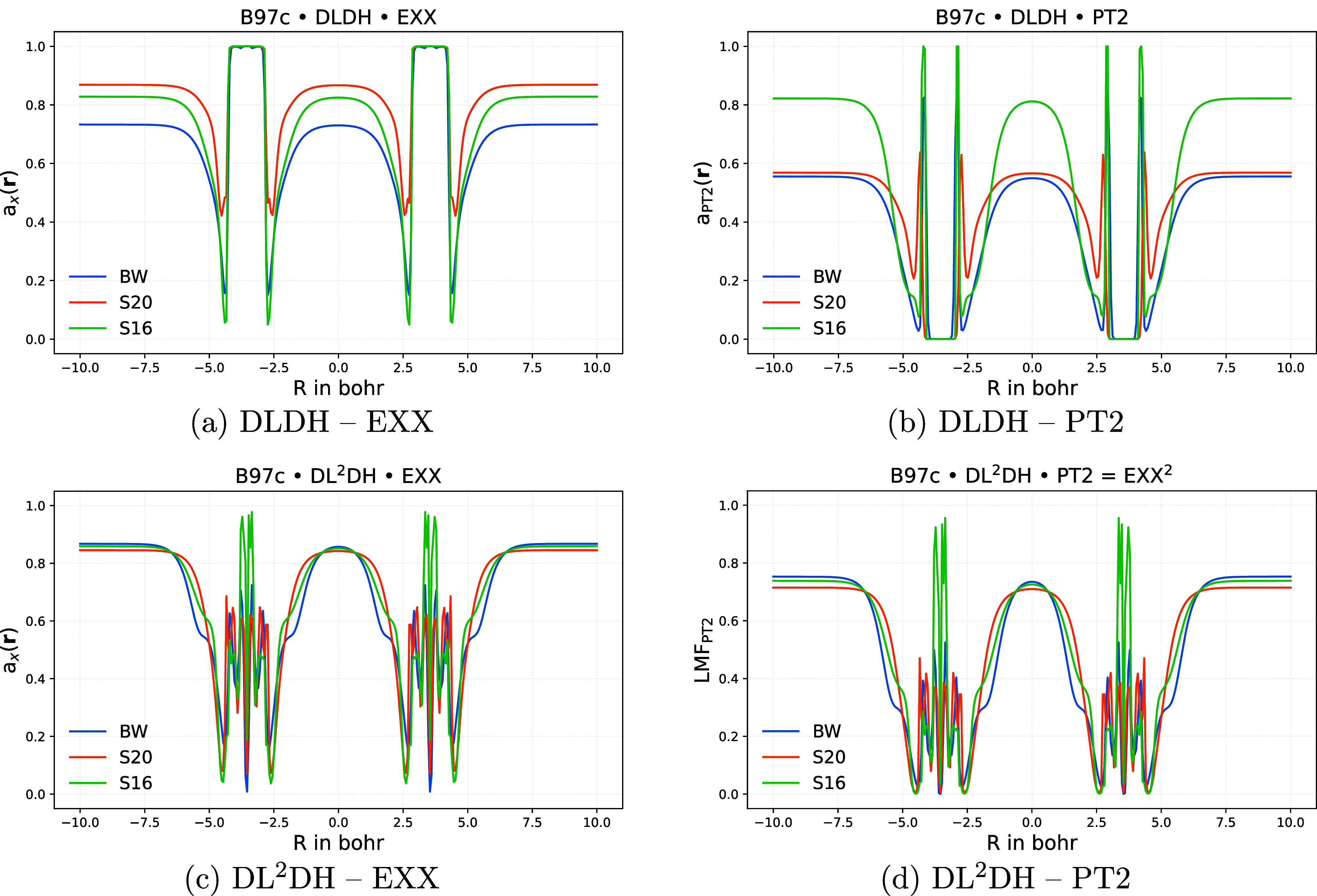
Plots of *a*_X_(**r**) (a, c) and *a*_PT2_(**r**) (b, d) along the bond axis for the Ar_2_ complex at an internuclear distance of 7.5 bohr and at epoch 800 for DLDH (a,b) and DL^2^DH functionals (c, d) trained with B97c using different training sets. Abbreviations: BW = BH76/W4–17 training, S16 = BH76/W4–17/Slim16 training, S20 = BH76/W4–17/Slim20 training.

We first note that all plots exhibit large values of both LMFs around the bond critical point (BCP) at the center of this NCI. For *a*_X_(**r**), this behavior is characteristic of LMFs that successfully suppress gauge artifacts in NCIs without the need for a calibration function.^10,11,49^ Similarly, the high values of *a*_PT2_(**r**) in this region likely contribute to the good NCI performance of both DLDH and DL^2^DH functionals without requiring additional dispersion corrections (see above). The LMF values in both BCP and the asymptotic regions are more stable with respect to training-data variations when the LMFs are coupled, as in the DL^2^DH model. In contrast, for DLDH functionals, training on BH76/W4–17/Slim16 yields the highest *a*_PT2_(**r**) values in both regions, while inclusion of either Slim16 or Slim20 tends to increase *a*_X_(**r**).

### 4.5 Argon–Benzene Dimer

As an example of a challenging potential-energy curve for a dispersion-dominated NCI, we examine the argon–benzene complex. Figure 8 shows representative results for the B97c-based functionals. This system has already been studied with various functionals.^66^ That study emphasized that many of the Minnesota functionals yield poor potential-energy curves due to their inadequate treatment of the long-range *r* ^–6^ dispersion interaction, and that the nonlocal VV10 van der Waals contribution,^72^ as included in functionals like B97M-V or ωB97M-V, performs notably better than standard atom-additive dispersion corrections for this case.

**Figure 8:**
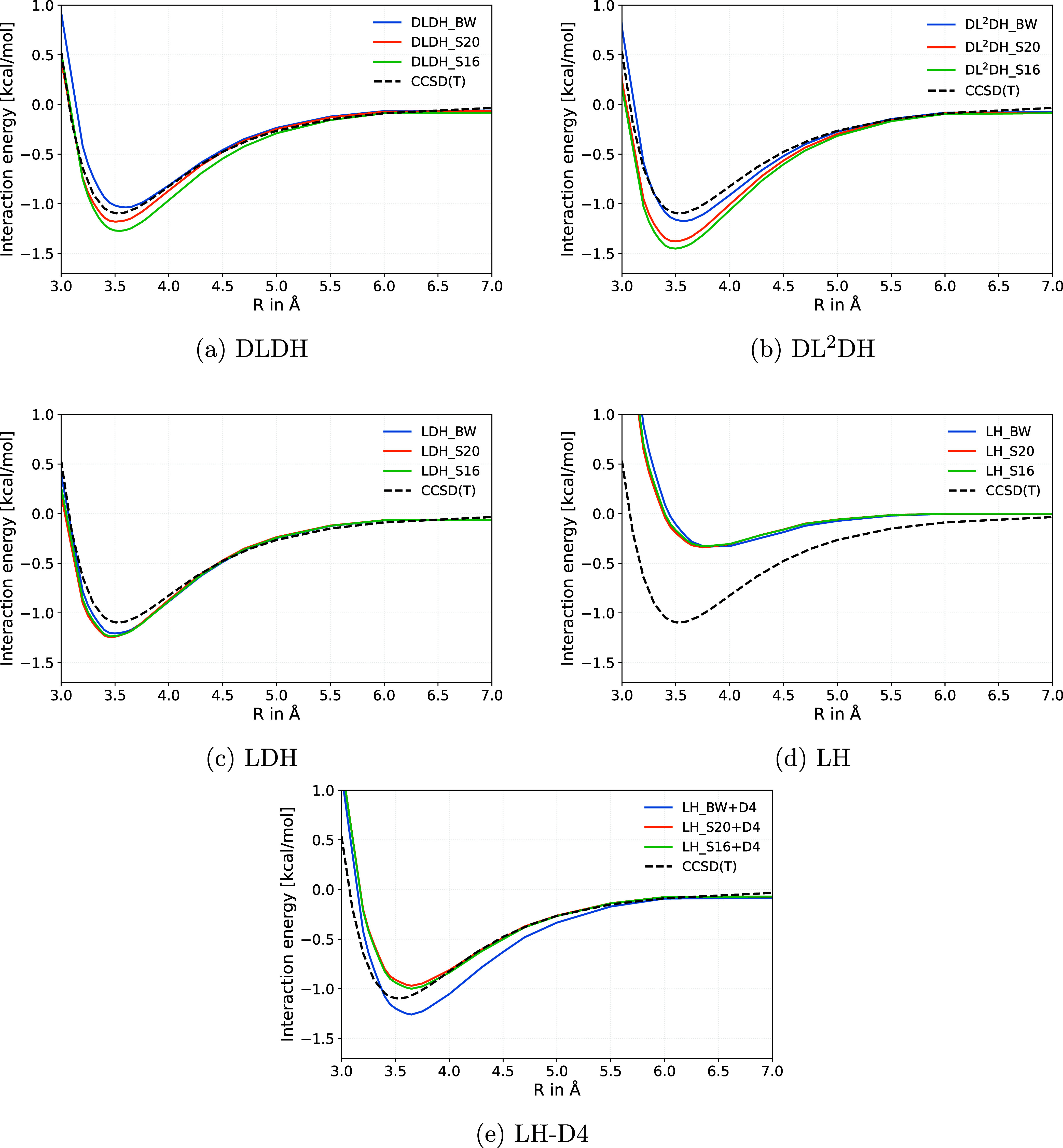
Comparison of (a) DLDH, (b) DL^2^DH, (c) LDH, (d) LH, and (e) LH-D4 dissociation curves of the argon–benzene complex (in C_6v_ symmetry, with B97c) calculated in PySCF with grid (99, 590) compared to the CCSD­(T)/aug-cc-pVQZ reference curve.^71^ Abbreviations: BW = BH76/W4–17 training, S16 = BH76/W4–17/Slim16 training, S20 = BH76/W4–17/Slim20 training.

Given the grid sensitivity of these curves (see Section 3), we present results obtained using the large numerical grid recommended in ref 66. Additional tests using the coarser “grid 1” setup employed during LMF training are provided in Supporting Information (Figures S8–S10) to assess the grid dependence of the n-LMF-based functionals.

The DLDH and DL^2^DH functionals trained on BH76/W4–17 closely match the CCSD­(T)/aug-cc-pVQZ reference curve^71^ (panels a and b on top). For the DLDHs (panel a), the LMFs obtained with the BH76/W4–17/Slim20 and BH76/W4–17 training protocols bracket the reference curve very well, whereas the BH76/W4–17/Slim16-trained LMF lies a bit lower. For the DL^2^DHs (panel b) the variants trained with inclusion of either Slim16 or Slim20 subsets overestimate binding moderately, while the LMF trained only on BH76/W4–17 comes closest to the reference, with a slightly too large equilibrium distance. All LDH curves (panel c) are closely parallel to the reference curve but also overstabilize slightly. Not unexpectedly, results for the LHs show that in contrast to the DLDH and LDH functionals, inclusion of dispersion corrections is mandatory to achieve agreement with the reference curve: without D4 corrections only very shallow minima are obtained (panel d), whereas after inclusion of D4 terms reasonable agreement is found, albeit still worse than with DLDH or DLH functionals. Equilibrium distances tend to be slightly too long, and binding energies may be either under- or overestimated for the LH, depending on whether Slim16 or Slim20 data were included in the training of *a*_*X*_(**r**).

Figure 9 provides a comparison of the DLDH and DL^2^DH curves (B97c variant with BH76/W4–17-only training data) to results with some literature functionals, in particular with the VV10-based ωB97M-V RSH^66^ and its meta-GGA cousin B97M-V.^73^ We also compare to the XYG3 xDH^14^ and to M06 ^74^ as an example of a Minnesota GH. An identical computational protocol (see above) was used in all cases. The previous finding of poor performance and numerical instability of M06 ^66^ is confirmed, showing that a mere fitting of a GH functional form without appropriate dispersion terms to NCI systems at their equilibrium structures is not a good recipe for reproducing such potential-energy curves. The DLDH and DL^2^DH models are competitive with the VV10-based functionals and the XYG3 DH. They bracket the reference curve most closely and give a very good equilibrium structure. XYG3 gives the closest agreement with the binding energy but a too short equilibrium distance. Additionally, to provide a clearer assessment of the deviations from the CCSD­(T)/aug-cc-pVQZ reference, Figure 10 shows a comparison of the equilibrium distances and binding energies obtained with the above-mentioned functionals. The dissociation curves for the Ar–benzene complex obtained with the approximately coupled DL^*ac*^DH functionals are provided in the Supporting Information (Figure S11).

**Figure 9:**
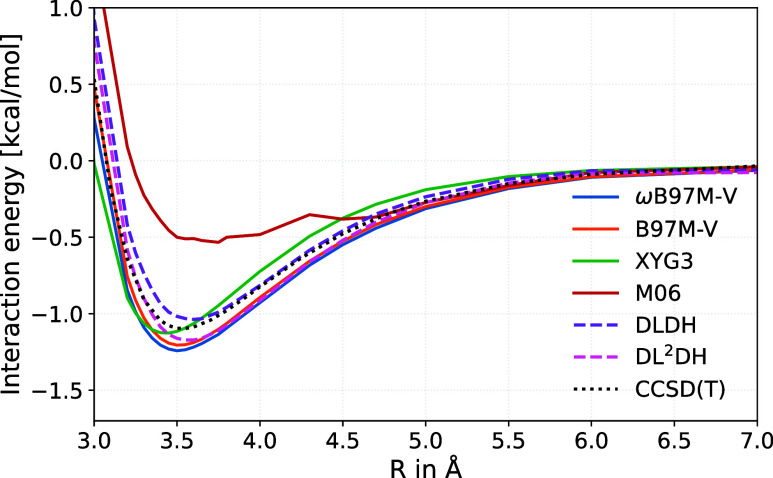
Comparison of benchmark functionals (ωB97M-V, B97M-V, M06, and XYG3) with the newly developed DLDH and DL^2^DH functionals for the argon–benzene interaction curve. The reference CCSD­(T)/aug-cc-pVQZ curve is shown as a dotted black line, while the DLDH and DL^2^DH functionals (trained on the BH76/W4–17 data set with B97 correlation) are plotted as dashed colored lines.

**Figure 10:**
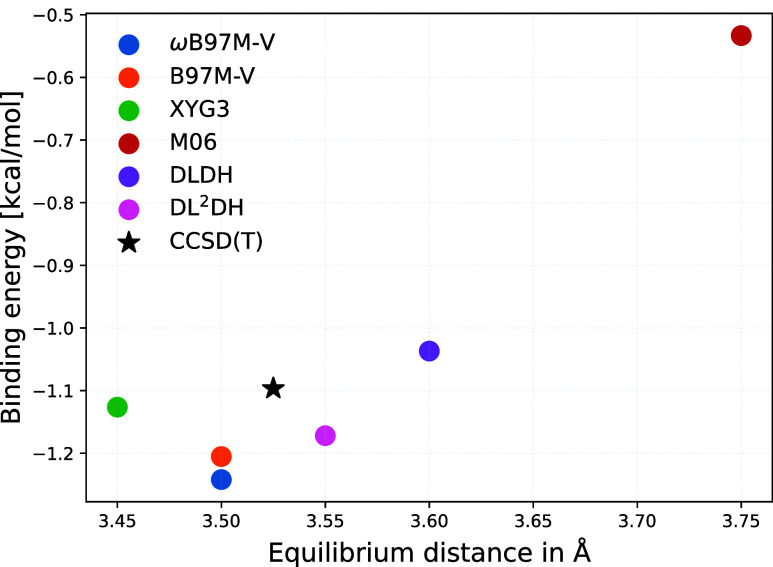
Binding energy vs equilibrium distance for the argon–benzene interaction for different functionals. The DLDH and DL^2^DH functionals are those trained on the BH76/W4–17 data set with B97 correlation.

We note in passing that the current inefficiency of computation of the PT2 energy density prevents us from evaluating larger grids for the entire test sets. However, our experience with n-LMFs so far for LHs and DLHs^10,11,31,32^ indicates numerically stable SCF procedures and moderate grid dependencies. This does not apply generally to machine-learned functionals. We suspect that our more limited use of neural networks to just shape the LMFs in otherwise well-defined functional forms leads to less numerical sensitivity.

## 5 Conclusions

This work extends the concept of double hybrid functionals to doubly local double hybrids (DLDHs), building on our recently proposed local double hybrids (LDHs). While DHs have constant, global admixtures of exact exchange (EXX) and PT2 correlation, LDHs combine position-dependent EXX admixture with constant PT2 contributions, and DLDHs finally use position-dependent EXX *and* PT2 admixtures in the most flexible combination. As we previously found it difficult to manually optimize the EXX local mixing function (LMF) for LDHs, we have utilized neural networks to construct and optimize the LMFs for both exchange and correlation. We used a neural network to either obtain two largely independent LMFs for the two contributions, or alternatively, restricted the correlation-LMF to be the square of the EXX-LMF to get a set of functionals we call DL^2^DHs (as suggested by arguments for “non-empirical double hybrids” based on the adiabatic connection and Görling-Levy perturbation theory).

As DLDHs require the PT2 correlation energy densities, and we currently have only access to a relatively inefficient implementation of such quantities, this work must be viewed as an initial exploration of the capabilities of DLDHs. We have not yet proposed any final production functional. Given the restriction of the training and evaluation data to molecules having up to about 20 atoms, we expect that the reported results of this work provide only a glimpse of the capability of such functionals when they are fully optimized. However, the DLDHs obtained here do indeed already provide moderate yet notable improvements over DHs or LDHs (and over LHs) constructed and optimized in the same manner. We note in particular, that both DLDHs and DL^2^DHs provide excellent results for dispersion-dominated noncovalent interactions without the need to add empirical dispersion corrections. This is found for the Slim16-GMTKN55 and Slim20-GMTKN55 evaluation sets, as well as for examples like the argon-benzene dissociation curve. The good performance without dispersion corrections can be traced to large PT2 contributions exhibited by the trained PT2 LMF near the bond-critical points of such noncovalent interactions. At the same time, the exchange LMFs also exhibit large EXX contributions in such regions, as well as in covalent bonds. This ensures a good suppression of unphysical artifacts arising from the gauge ambiguity of exchange-energy densities without the need to introduce a separate calibration function. LDHs exhibit less flexibility than DLDHs and require large EXX admixtures over large regions of space.

The promising results of this initial study indicate that efficient implementations of PT2 correlation-energy densities into fast computer codes may indeed be well worth the effort. Work in this direction is planned in our laboratories. We note that here we have not at all attempted to include any strong-correlation terms into the form of the LMF nor explicitly strong-correlation cases into the training data. Local hybrids and range-separated local hybrids have recently been shown to be capable of escaping to a significant extent the so-called zero-sum game between small delocalization and static-correlation errors. This is due to the flexibility of position-dependent EXX admixture. We expect that these advantages may also transfer to functionals like the present DLDHs. Of course, construction of such functionals with favorable properties in that area will also require the use of modified descriptions of the nonlocal correlation contributions that do not diverge for strong-correlation cases or small to vanishing band gaps. This may lead to very interesting new types of functionals on rung 5. Furthermore, lower-rung surrogates for such nonlocal correlation contributions might also be found by machine learning techniques. This would further enhance the potential of such approaches.

## Supplementary Material



## Data Availability

The neural network training code for LMFs and the code for the generation of PT2 and exact-exchange energy densities is publicly available at https://github.com/norakovacs98/Doubly-Local-Double-Hybrid.git.

## Notes

Detailed benchmarking data, optimized D4 dispersion parameters, and analyses of local mixing functions and dissociation curves for DLDH, DL^2^DH, and “approximately coupled” DL^*ac*^DH variants (PDF)
