# Supplementary material for: Toward Doubly Local Double Hybrid Functionals Using Neural-Network Local Mixing Functions
Source: J Chem Theory Comput. 2026 Feb 13;22(7):3268–81. doi: 10.1021/acs.jctc.5c01952 (PMC13085251; doi:10.1021/acs.jctc.5c01952)
Supplement: Supplementary file 1 [file ct5c01952_si_001.pdf]

Supplementary Information: Towards doubly local double hybrid functionals using neural-network local mixing functions.

Nóra Kovács,[†] Szymon Śmiga,[‡] Martin Kaupp,[*,†] and Artur Wodyński[*,†]

[†]*Technische Universität Berlin, Institut für Chemie, Theoretische Chemie/Quantenchemie, Sekr. C7, Straße des 17. Juni 135, D-10623, Berlin, Germany*

[‡]*Institute of Physics, Faculty of Physics, Astronomy and Informatics, Nicolaus Copernicus University in Toruń, ul. Grudziądzka 5, 87-100 Toruń, Poland*

E-mail: martin.kaupp@tu-berlin.de; artur.wodynski@tu-berlin.de

S1 Implementation details

Neural-network local mixing functions (n-LMFs). In this work, the local mixing functions are constructed as a multi-layer perceptron (MLP). At each grid point, the vector of input features consists of seven local quantities:

$$x(\mathbf{r}) = \big(\rho_\alpha,\ \rho_\beta,\ |\nabla\rho_\alpha|^2,\ \nabla\rho_\alpha \cdot \nabla\rho_\beta,\ |\nabla\rho_\beta|^2,\ \tau_\alpha,\ \tau_\beta\big) \in \mathbb{R}^7.$$

To keep values in a numerically advantageous range, each feature is logarithmically rescaled individually:

$$x_s(\mathbf{r}) = \mathrm{sgn}(x(\mathbf{r})) \cdot \log\!\big(1 + |x(\mathbf{r})|\big).$$

The rescaled inputs are passed through an MLP with three hidden layers of 128 neurons each. The hidden layers employ the GeLU activation function,[52]

$$\mathrm{GeLU}(x) = \tfrac{x}{2}\left[1 + \mathrm{erf}\!\left(\tfrac{x}{\sqrt{2}}\right)\right], \tag{S1}$$

while the final layer applies a standard sigmoid function[?] to map the output to $(0, 1)$:

$$\sigma(x) = \frac{1}{1 + e^{-x}}. \tag{S2}$$

For the standard MLP architectures with a single output neuron (for DL^{2}DHs, LDHs, and LHs), this reads explicitely:

$$\begin{aligned}
h_1(\mathbf{r}) &= \mathrm{GeLU}(W_1 x_s(\mathbf{r}) + b_1), & W_1 &\in \mathbb{R}^{128\times 7},\ b_1 \in \mathbb{R}^{128},\\
h_2(\mathbf{r}) &= \mathrm{GeLU}(W_2 h_1(\mathbf{r}) + b_2), & W_2 &\in \mathbb{R}^{128\times 128},\ b_2 \in \mathbb{R}^{128},\\
h_3(\mathbf{r}) &= \mathrm{GeLU}(W_3 h_2(\mathbf{r}) + b_3), & W_3 &\in \mathbb{R}^{128\times 128},\ b_3 \in \mathbb{R}^{128},\\
\tilde{a}(\mathbf{r}) &= \sigma(W_4 h_3(\mathbf{r}) + b_4), & W_4 &\in \mathbb{R}^{1\times 128},\ b_4 \in \mathbb{R}.
\end{aligned}$$

For the DLDH functional with two to some extent independent LMFs, the output layer instead contains two neurons,

$$\tilde{a}(\mathbf{r}) = \sigma(W_4 h_3(\mathbf{r}) + b_4), \quad W_4 \in \mathbb{R}^{2\times 128},\ b_4 \in \mathbb{R}^2,$$

corresponding to the two LMFs $a_{\mathrm{x}}(\mathbf{r})$ for exchange and $a_{\mathrm{PT2}}(\mathbf{r})$ for correlation. With this setup, the total number of trainable parameters is 34,177 for the DL^{2}DH, LDH and LH functionals, and 34,306 for the DLDH functional. To enforce invariance under exchange of spin labels, the network is evaluated twice, once with (α, β) ordering and once with (β, α), and the results are averaged:

$$a_{\mathrm{n}}(\mathbf{r}) = \tfrac{1}{2}\Big[\tilde{a}\big(x_s^{(\alpha,\beta)}(\mathbf{r})\big) + \tilde{a}\big(x_s^{(\beta,\alpha)}(\mathbf{r})\big)\Big].$$

Model exchange-energy density. The chosen semi-local exchange contribution is based on the PBE form:[53]

$$e_X^{\mathrm{PBE}}(\mathbf{r}) = -C_X \rho^{4/3}(\mathbf{r}) \left(1 + \kappa - \frac{\kappa}{1 + \mu s^2/\kappa}\right), \tag{S11}$$

where

$$C_X = \tfrac{3}{4}\left(\tfrac{6}{\pi}\right)^{1/3}, \quad s^2 = \frac{|\nabla\rho(\mathbf{r})|^2}{4 \cdot 3^{2/3}\pi^{4/3}\rho^{8/3}(\mathbf{r})}, \quad \mu = 0.21951, \quad \kappa = 0.804.$$

For spin-polarized cases, the spin-scaling relation is applied to obtain the total exchange energy:

$$e_X^{\mathrm{PBE}}(\rho_\sigma, \rho_{-\sigma}) = \frac{e_X^{\mathrm{PBE}}(2\rho_\sigma) + e_X^{\mathrm{PBE}}(2\rho_{-\sigma})}{2}. \tag{S12}$$

Semi-local correlation models. The B95c[54] and B97c[55] meta-GGA correlation functionals can both be written in terms of linear coefficients d and nonlinear parameters c, applied separately to opposite-spin and same-spin channels. The specific parameter values used in this work are summarized in Table **??**. They have not been optimized in this initial

work on DLDHs, but the B95c parameters have been taken from the LH20t functional[4] and the B97c parameters from the recent LH24n functional.[10]

For B95c, only a single linear coefficient per opposite- and same-spin correlation is used:

$$\begin{aligned} e_c^{\mathrm{B95}}(\mathbf{r}) = d_{opp}\left[1 + c_{opp}(\chi_\alpha^2(\mathbf{r}) + \chi_\beta^2(\mathbf{r}))\right]^{-1} e_{c,opp}^{\mathrm{UEG}}(\mathbf{r}) \\ + d_{\sigma\sigma} \sum_{\sigma=\alpha,\beta} \left[1 + c_{\sigma\sigma}\chi_\sigma^2(\mathbf{r})\right]^{-2} \alpha_\sigma(\mathbf{r})\, e_{c,\sigma\sigma}^{\mathrm{UEG}}(\mathbf{r}). \end{aligned} \tag{S1}$$

For B97c, this structure is generalized by using longer polynomial expansions in the reduced gradients:

$$e_c^{\mathrm{B97}}(\mathbf{r}) = \sum_i d_{opp,i}\, f_{opp}(\chi_\alpha, \chi_\beta)\, e_{c,opp}^{\mathrm{UEG}}(\mathbf{r}) + \sum_{\sigma=\alpha,\beta} \sum_i d_{\sigma\sigma,i}\, f_{\sigma\sigma}(\chi_\sigma)\, \alpha_\sigma(\mathbf{r})\, e_{c,\sigma\sigma}^{\mathrm{UEG}}(\mathbf{r}), \tag{S2}$$

with

$$f_{opp}(\chi_\alpha, \chi_\beta) = \frac{c_{opp}(\chi_\alpha^2 + \chi_\beta^2)}{1 + c_{opp}(\chi_\alpha^2 + \chi_\beta^2)}, \tag{S3}$$

$$f_{\sigma\sigma}(\chi_\sigma) = \frac{c_{\sigma\sigma}\chi_\sigma^2}{1 + c_{\sigma\sigma}\chi_\sigma^2}. \tag{S4}$$

$d_{opp,i}$ and $d_{\sigma\sigma,i}$ are the linear expansion coefficients given in Table **??**, while c_{opp} and $c_{\sigma\sigma}$ are shared nonlinear parameters.

Table S1. Linear and nonlinear parameters employed in the B95c and B97c correlation models.

**Model**	**Parameter**	**Values**
B95c[a]	d_{opp}	1.2291
	$d_{\sigma\sigma}$	0.8178
	c_{opp}	0.0050
	$c_{\sigma\sigma}$	0.0954
B97c[b]	$d_{opp,i}$	1.4014, -2.9021, 3.4140, -1.8361
	$d_{\sigma\sigma,i}$	0.2288, -0.5282, 0.9106, -0.6228
	c_{opp}	0.0050
	$c_{\sigma\sigma}$	0.0954

[a]Parameters taken from Ref. 4. [b]Parameters taken from Ref. 10.

Dispersion parameters.

Table S2. D4 Dispersion parameters (s_6, s_8, a_1, a_2)? for the B95c-trained functionals.

Functional	Training set	s_6	s_8	a_1	a_2
DLDH	BW	0.2000	0.2774	0.8068	3.1975
DL^2DH	BW	0.3368	0.3088	0.9231	3.0586
LDH	BW	0.0880	0.0278	0.7656	3.0347
LH	BW	0.9991	0.0361	0.6438	2.9841
DLDH	S20	0.1044	0.0568	0.7838	3.0384
DL^2DH	S20	0.0141	-0.0056	0.7587	3.0330
LDH	S20	0.4000	-0.4267	1.1306	3.2665
LH	S20	1.0000	2.0000	1.0488	3.0600
DLDH	S16	0.1353	0.0844	0.7945	3.0412
DL^2DH	S16	0.1326	0.0696	0.7844	3.0388
LDH	S16	0.3000	0.1883	1.0783	3.2295
LH	S16	1.0000	2.0000	0.8804	3.6772

Table S3. D4 Dispersion parameters (s_6, s_8, a_1, a_2)? for the B97c-trained functionals.

Functional	Training set	s_6	s_8	a_1	a_2
DLDH	BW	0.4000	0.1359	0.8736	3.2058
DL^2DH	BW	0.2382	0.1117	0.6973	2.7242
LDH	BW	0.4000	0.2917	0.9878	3.2121
LH	BW	1.0000	1.3951	0.6115	4.1731
DLDH	S20	0.4000	-0.1742	1.5053	3.3463
DL^2DH	S20	0.0255	-0.0008	0.7645	3.0344
LDH	S20	0.5000	0.0012	1.3545	3.2905
LH	S20	1.0000	0.5810	0.8552	3.0793
DLDH	S16	0.0149	-0.2910	1.6071	3.3741
DL^2DH	S16	0.1199	0.0459	0.7795	3.0378
LDH	S16	0.4000	0.3233	1.1922	3.2469
LH	S16	1.0000	0.6006	0.8341	3.0603

S2 Benchmarking of initial training setups

For initial exploration of the impact of input orbitals, neural-network architecture and PT2 admixture on performance, we first carried out simulations on the W4–17 and BH76 test sets using B95c correlation. Table **??** summarizes mean absolute errors (MAEs) for all four types of functionals (DLDH, DL^2DH, LDH, and LH), trained on PBE orbitals containing 25%, 60%, or 80% EXX admixture. Results are reported at training epochs 800, 1200, and 2000.

Table S4. Mean absolute errors (MAE, in kcal/mol) on the W4-17 and BH76 test sets at epochs 800, 1200 and 2000, for various forms of functionals with B95c. The "Input orbital" column indicates the PBE-based GHs used to generate the input orbitals, with 25%, 60% or 80% EXX mixing.

Category	Variant	Input orbitals (% EXX)	Epoch 800		Epoch 1200		Epoch 2000	
			W4-17	BH76	W4-17	BH76	W4-17	BH76
DLDH		25	1.259	0.674	1.156	0.607	1.111	0.464
		60	1.086	0.628	1.069	0.782	1.034	0.564
		80	1.364	1.100	1.334	0.897	1.302	0.818
DL^2DH		25	7.388	3.468	7.538	3.800	5.750	2.723
		60	1.264	0.916	1.312	0.788	0.947	0.550
		80	1.668	0.951	1.516	0.886	1.458	0.676
LDH	$c_{PT2} = 0.3$	25	18.995	2.333	18.995	2.333	18.995	2.333
	$c_{PT2} = 0.4$	25	30.990	4.438	30.990	4.438	30.990	4.438
	$c_{PT2} = 0.5$	25	42.991	6.595	42.991	6.595	42.991	6.595
	$c_{PT2} = 0.6$	25	54.987	8.752	54.987	8.752	54.987	8.752
	$c_{PT2} = 0.7$	25	66.977	10.907	66.977	10.907	66.977	10.907
	$c_{PT2} = 0.3$	60	1.953	1.009	1.476	0.765	1.256	0.428
	$c_{PT2} = 0.4$	60	1.242	0.893	1.291	0.809	1.050	0.416
	$c_{PT2} = 0.5$	60	6.726	2.884	6.726	2.884	6.726	2.884
	$c_{PT2} = 0.6$	60	10.340	2.160	10.340	2.160	10.340	2.160
	$c_{PT2} = 0.7$	60	17.491	2.156	17.491	2.156	17.491	2.156
	$c_{PT2} = 0.3$	80	1.550	1.161	1.607	1.014	1.354	0.573
	$c_{PT2} = 0.4$	80	1.510	1.157	1.581	1.046	1.352	0.645
	$c_{PT2} = 0.5$	80	1.747	1.393	1.497	1.256	1.285	0.521
	$c_{PT2} = 0.6$	80	1.621	1.290	1.469	0.765	1.255	0.347
	$c_{PT2} = 0.7$	80	1.832	1.230	1.382	1.005	1.797	0.853
LH		25	2.194	0.830	1.805	0.735	1.554	0.511
		60	2.077	0.826	1.908	0.888	1.699	0.790
		80	2.118	1.015	1.951	0.893	1.936	0.856

Table S5. Comparison of mean absolute errors (MAE, in kcal/mol) on the W4-17 and BH76 test sets at epoch 800 for two neural-network architectures: (i) 128 neurons with three hidden layers and (ii) 64 neurons with two hidden layers. Results are shown for DLDH and DL^2DH functionals, using input orbitals from PBE-based GHs with 25%, 60%, and 80% EXX admixture.

Category	Input orbitals	128 neurons / 3 layers		64 neurons / 2 layers	
	(% EXX)	W4-17	BH76	W4-17	BH76
DLDH	25	1.259	0.674	1.371	0.754
	60	1.086	0.628	5.089	2.450
	80	1.364	1.100	1.353	1.051
DL^2DH	25	7.388	3.468	5.904	3.736
	60	1.264	0.916	1.242	1.027
	80	1.668	0.951	1.723	0.972

Table S6. Comparison of mean absolute errors (MAE, in kcal/mol) on the W4-17 and BH76 test sets at epoch 2000 for two neural-network architectures: (i) 128 neurons with three hidden layers and (ii) 64 neurons with two hidden layers. Results are shown for DLDH and DL^2DH functionals, using input orbitals from PBE-based GHs with 25%, 60%, and 80% EXX admixture.

Category	Input orbitals	128 neurons / 3 layers		64 neurons / 2 layers	
	(% EXX)	W4-17	BH76	W4-17	BH76
DLDH	25	1.111	0.464	1.203	0.597
	60	1.034	0.564	5.037	2.076
	80	1.302	0.818	1.197	0.869
DL^2DH	25	5.750	2.723	5.447	2.740
	60	0.947	0.550	1.052	0.942
	80	1.458	0.676	1.639	0.935

S3 Training losses (MAEs) at epoch 800 for different training sets

Tables ??–?? report MAEs (in kcal/mol) corresponding to the loss components evaluated during training at epoch 800 for all four classes of functionals (DLDH, DL^2DH, LDH, and LH), using either B95c or B97c correlation. Results are shown for three training set combinations (BH76/W4-17, BH76/W4-17/Slim16, and BH76/W4-17/Slim20). "MAE_{av}" averages the deviations for the two test sets with equal weights.

Table S7. Training losses (mean absolute errors, MAEs, in kcal/mol) at epoch 800 for the four classes of functionals trained on the combined BH76/W4-17 dataset, using B95c or B97c correlation. These values correspond to the MAE components of the total loss function during training

	B95c				B97c			
Testset	DLDH	DL^2DH	LDH	LH	DLDH	DL^2DH	LDH	LH
BH76	0.828	0.974	0.893	0.827	1.058	0.963	1.367	1.091
W4-17	1.086	1.315	1.242	2.077	1.234	1.195	2.103	2.496
MAE_{av}	0.957	1.220	1.068	1.452	1.146	1.131	1.735	1.793

Table S8. Training losses (mean absolute errors, MAEs, in kcal/mol) at epoch 800 for the four classes of functionals trained on the combined BH76/W4-17/Slim16 dataset, using B95c or B97c correlation. These values correspond to the MAE components of the total loss function during training

	B95c				B97c			
Testset	DLDH	DL^2DH	LDH	LH	DLDH	DL^2DH	LDH	LH
ADIM6	0.2772	0.3059	0.4974	1.1868	0.3250	0.2963	0.4687	1.1389
AHB21	0.6699	0.7240	0.7417	0.4474	0.6268	0.7791	0.7408	0.3057
AL2X6	0.6343	0.1317	0.0215	3.0855	0.4811	0.8977	0.2849	3.8515
ALK8	0.2300	0.5938	0.3254	0.6126	0.4789	0.9385	0.6130	0.3257
ALKBDE10	1.5253	1.0609	2.6313	0.2508	2.1860	1.7647	1.6019	0.1551
BH76	0.3073	0.7744	0.7645	0.6366	0.6132	0.9455	1.2456	0.9796
BH76_full	1.0510	1.0428	1.3076	1.3775	1.0878	1.0629	1.5474	1.5419
BHDIV10	1.8405	2.2426	1.1702	0.2510	1.0744	1.9171	0.0212	0.9938
BHPERI	1.0667	0.9495	1.4833	2.4497	1.3301	1.5431	1.9859	2.0787
BHROT27	0.0305	0.0640	0.5906	0.6098	0.1023	0.0669	0.3561	0.5859
BUT14DIOL	0.0744	0.0883	0.0400	0.1388	0.0717	0.0666	0.0469	0.1199
CARBHB12	0.3491	0.3874	0.2725	0.3108	0.3874	0.4640	0.3108	0.3491
DC13	8.1886	7.5477	5.3574	10.0923	6.6931	7.0875	4.6726	8.3017
DIPCS10	2.1403	1.3121	2.0829	6.1373	0.6227	2.4084	1.0673	6.6831
FH51	0.3697	0.4056	0.3018	1.0065	0.3099	0.2548	0.3377	0.8748
G2RC	0.9161	0.7416	1.9553	3.5591	0.4719	0.3443	1.9984	3.3634
G21EA	2.8373	1.1306	4.8146	2.5070	4.4890	2.3370	4.1778	3.1150
G21IP	1.4243	0.0359	1.0109	2.1520	1.3094	0.8306	1.4705	1.7307
HAL59	0.2025	0.1307	0.2231	0.3918	0.2145	0.0781	0.2758	0.4266
HEAVY28	0.0260	0.0483	0.0598	0.6786	0.0293	0.0304	0.0933	0.7157
ICONF	0.0212	0.0212	0.2852	0.7448	0.0212	0.0212	0.5916	0.7448
IL16	0.8672	0.7906	0.6451	2.1681	0.6738	1.0395	0.7025	2.1681
ISO34	0.7510	0.2244	1.1244	0.7079	0.5020	0.2196	1.3925	1.2728
MB1643	6.1606	7.1762	3.8528	12.0313	3.8787	6.0235	8.2160	16.9577
PA26	0.6019	0.6879	0.7569	0.4285	0.6799	0.5870	0.9013	0.9392
PNICO23	0.0508	0.3572	0.7152	2.7068	0.1024	0.3572	0.6386	2.5536
PX13	0.8506	0.9655	0.9774	2.2762	0.7827	0.6272	1.1626	3.2432
RC21	1.7936	1.4417	2.5021	2.2723	0.5536	0.8145	2.8827	2.2675
RG18	0.1615	0.1216	0.1391	0.4152	0.1678	0.1088	0.1902	0.4631
RSE43	0.0389	0.0689	0.6122	0.4519	0.1550	0.1552	0.4195	0.5058
S66	0.3043	0.3107	0.4096	1.1309	0.4160	0.2436	0.4543	1.1324
SIE4x4	1.0548	0.6730	1.3062	3.0494	1.4899	0.8903	1.0242	3.5804
TAUT15	0.1525	0.1433	0.2929	0.2427	0.1305	0.0348	0.0950	0.0283
W411	0.6827	0.9509	1.3463	1.6401	0.8863	0.7379	2.2465	2.2511
W4-17	1.6096	1.7036	1.9520	2.4968	1.2361	1.5444	3.0096	2.7707
WATER27	4.9402	4.7822	4.6003	4.4279	5.0934	4.7247	4.8731	4.1167
WCPT18	3.1859	2.8604	3.1859	2.0082	2.9466	2.6402	3.5306	0.0217
YBDE18	0.4593	0.3811	0.6862	1.2287	1.0110	0.4145	0.8717	1.4490
MAE_{av}	1.3523	1.4005	1.5993	2.1196	1.1390	1.3146	2.2889	2.3671

Table S9. Training losses (mean absolute errors, MAEs, in kcal/mol) at epoch 800 for the four classes of functionals trained on the combined BH76/W4-17/Slim20 dataset, using B95c or B97c correlation. These values correspond to the MAE components of the total loss function during training

	B95c				B97c			
Testset	DLDH	DL^2DH	LDH	LH	DLDH	DL^2DH	LDH	LH
ACONF	0.1735	0.0682	0.0490	0.3458	0.1448	0.0873	0.0873	0.4512
AHB21	1.9080	1.9511	1.6998	1.8410	1.8626	1.9248	1.6830	1.7501
ALKBDE10	3.0895	1.1553	2.9722	4.8561	3.1781	1.0572	3.2810	3.5443
AMINO20x4	0.0146	0.0428	0.0237	0.0146	0.0146	0.0529	0.0338	0.0338
BHPERI	2.1921	3.4592	2.9836	1.6635	2.2559	3.2549	2.4155	1.9284
BH76	2.1914	1.9900	2.1896	2.3547	2.2498	2.0672	2.3432	2.3399
BH76_full	1.4180	1.5301	2.0381	2.7414	1.4993	1.6272	1.9563	2.7654
BSR36	0.1038	0.5251	0.0016	3.5252	0.1133	0.2570	0.0111	3.4199
BUT14DIOL	0.0343	0.0358	0.0497	0.0640	0.0351	0.0547	0.0447	0.0640
CDIE20	0.7821	0.8363	0.3608	0.9756	0.4339	0.4307	0.3628	1.0043
FH51	0.3556	0.4086	0.7681	1.4318	0.3558	0.2417	0.6595	1.1798
G2RC	1.1497	1.3008	1.5806	2.8573	1.7749	1.2306	2.4966	3.1637
G21EA	2.3876	1.1237	2.5791	3.5558	2.6557	1.2578	3.1345	3.8622
G21IP	2.1424	1.0506	2.1019	3.9458	2.1229	1.3241	1.8182	4.0511
HAL59	0.2775	0.3521	0.2256	0.6965	0.2593	0.3656	0.2266	0.7980
HEAVY28	0.1929	0.1929	0.0783	0.9274	0.0783	0.2312	0.1423	1.0327
ICONF	0.0238	0.1294	0.0528	0.4358	0.0238	0.1294	0.0528	0.4358
IL16	0.6056	0.8584	0.3796	2.3984	0.5813	0.7090	0.3375	2.3142
INV24	0.5771	0.2272	1.5729	0.9409	0.7303	0.0932	1.8984	1.1516
ISO34	0.0956	1.1393	0.7850	1.2660	0.2468	1.1871	0.4211	0.9405
MB1643	9.0743	6.4316	17.2682	28.5220	6.2146	3.9790	16.6426	17.5044
PA26	0.1559	0.6676	0.5910	0.2516	0.9985	0.1942	0.1559	0.9410
PArel	0.0128	0.9895	0.0703	1.6023	0.1086	0.8554	0.0511	2.3300
PNICO23	0.1223	0.1472	0.0942	0.7342	0.0878	0.1903	0.1038	0.7629
PX13	0.2075	1.7778	0.3990	0.0160	0.8586	1.7395	0.8969	0.3287
RSE43	0.1652	0.2437	0.2093	0.5547	0.1088	0.1698	0.2069	0.5517
S22	0.2261	0.1495	0.2335	3.8337	0.0346	0.0037	0.3484	4.1018
S66	0.0851	0.0729	0.1064	0.7210	0.1423	0.0705	0.1354	0.8080
SIE4x4	2.0459	2.5737	0.1654	2.6824	1.7259	2.1046	0.0872	3.4476
TAUT15	0.0096	0.0287	0.0670	0.6798	0.1436	0.0670	0.1436	0.3351
W411	1.8648	2.6507	2.5410	3.1479	1.6444	2.4345	2.7040	4.0267
W4-17	2.9943	2.5390	3.3797	5.9700	2.2867	2.5214	3.2597	4.2507
WATER27	0.9354	0.7630	1.3886	3.0547	0.6034	0.9609	1.4109	2.8855
WCPT18	1.5544	1.6693	1.7459	2.6699	1.4060	1.5352	1.4299	2.1672
YBDE18	0.7498	0.6264	1.7277	2.3804	0.8077	0.9300	1.9915	1.7044
MAE_{av}	2.2065	1.9854	2.6466	4.4509	1.8195	1.9649	2.5701	3.4581

S4 Evolution of LMFs with training epochs

Figures **??**–**??** illustrate how the trained LMFs evolve for the CS molecule at different training epochs (400, 600, 800, 1200), using different choices of training sets. For BH76/W4-17 training (Figure **??**), the DLDH model shows a gradual decrease in the EXX admixture in the bonding region with a corresponding rise in the PT2 LMF. In contrast, DL^2DH changes much less between epochs 400 and 800, with noticeable adjustments only appearing at epoch 1200, particularly in the bonding region. The LH model is largely stable across epochs, apart from a higher EXX admixture in the asymptotic region at epoch 1200. We note again (see main text) that the training data in this work generally do not reflect the core nor asymptotic regions.

For BH76/W4-17/Slim16 training (Figure **??**), most functionals again show only modest changes across epochs. Two exceptions stand out: for DL^2DH, the EXX contribution in the bonding region decreases from over 20% at epoch 400 to below 10% at epoch 1200, while for the LDHs the EXX fraction drops from nearly 50% at epoch 400 to just above 20% at epoch 1200.

BH76/W4-17/Slim20 training (Figure **??**) leads to largely stable LMFs as well. The main exception is the LDH exchange channel, where the bonding-region EXX fraction decreases significantly from almost 40% at epoch 400 to about 10% at epoch 1200.

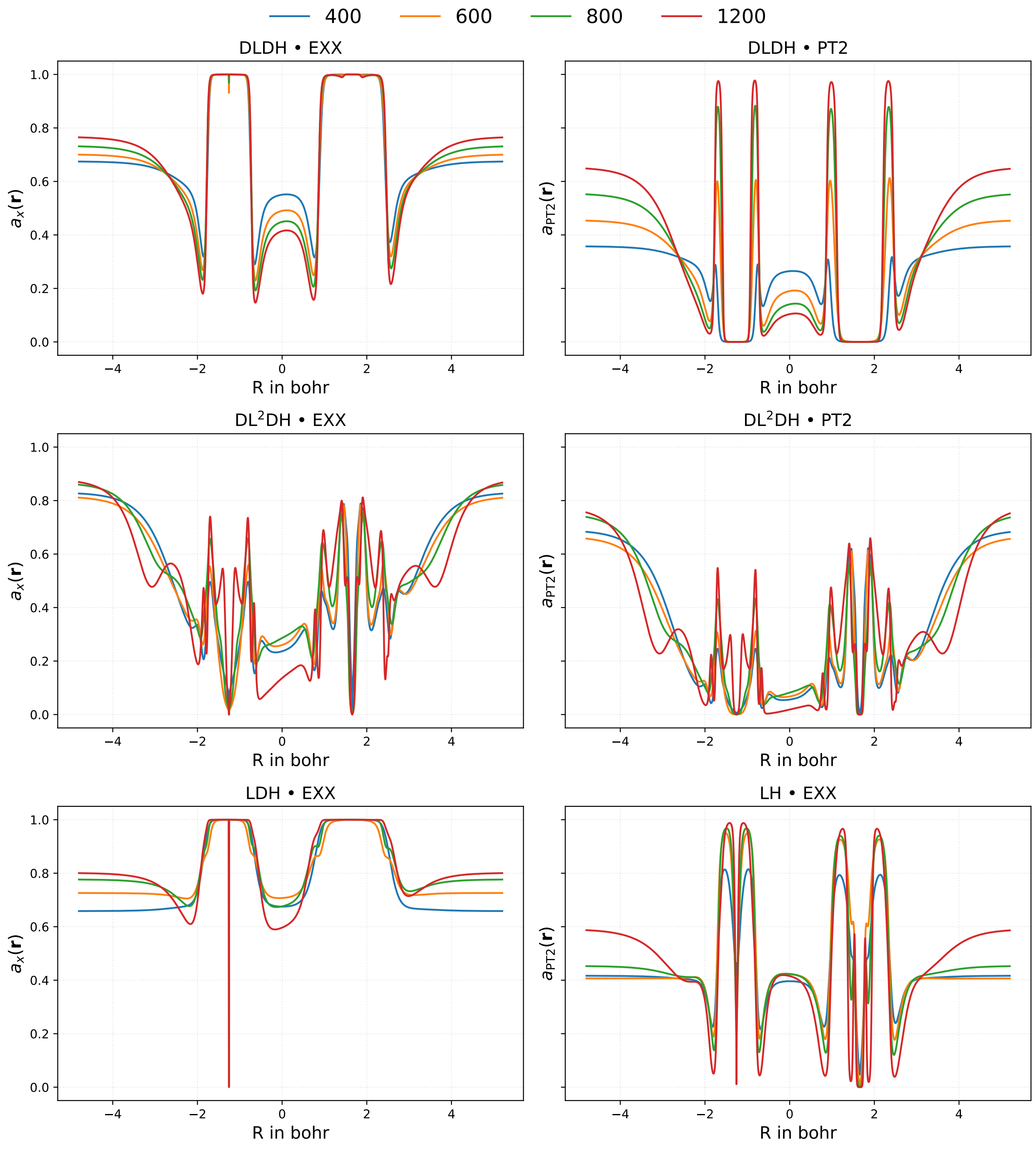

Figure S1. Evolution of LMFs for the CS molecule (with B97c) with training epoch for different types of functionals, upon training on BH76/W4-17. Panels show LMFs for epochs 400, 600, 800, and 1200.

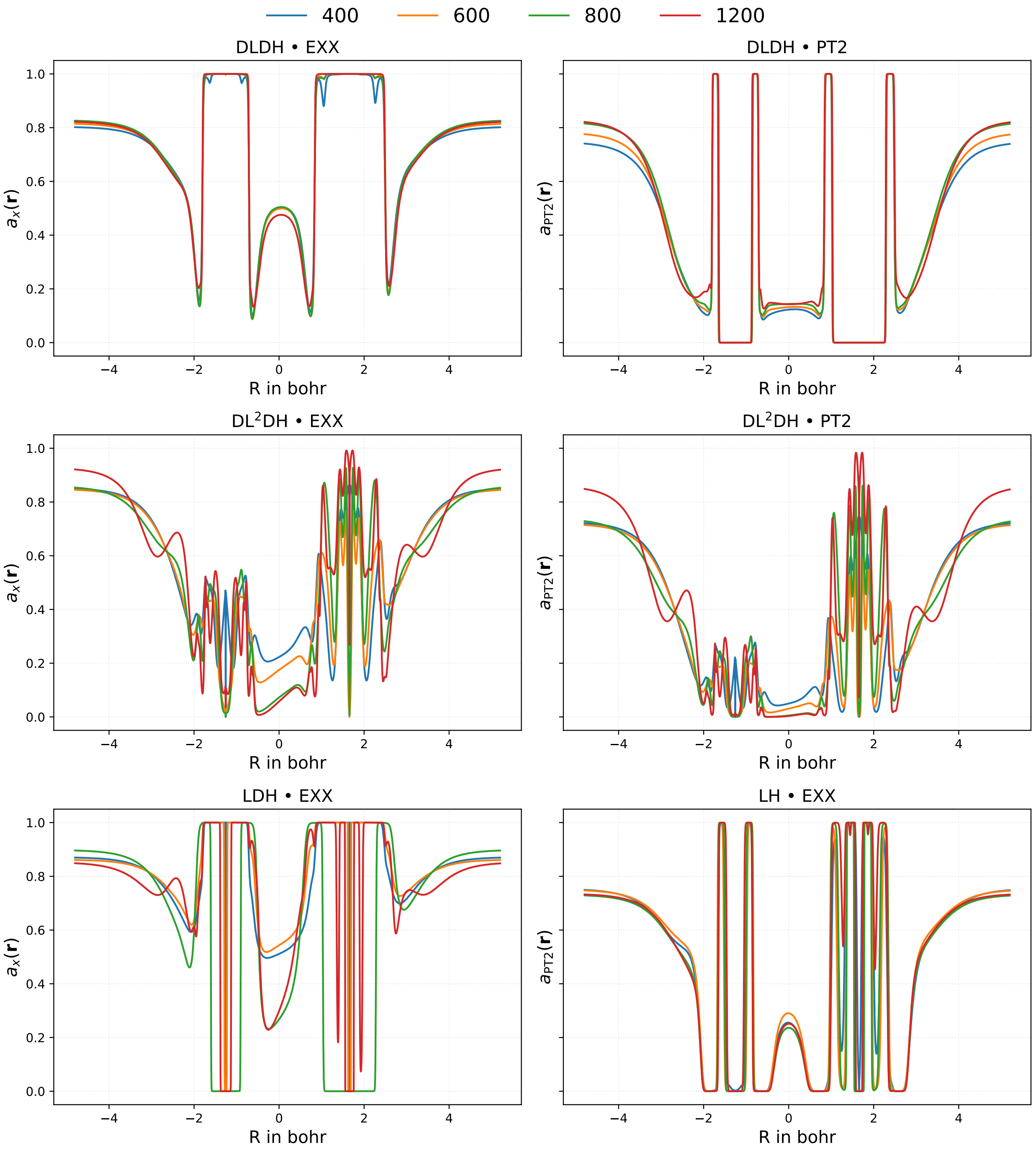

Figure S2. Evolution of LMFs for the CS molecule (with B97c) with training epoch for different types of functionals, upon training on BH76/W4-17/Slim16. Panels show LMFs for training epochs 400, 600, 800, and 1200.

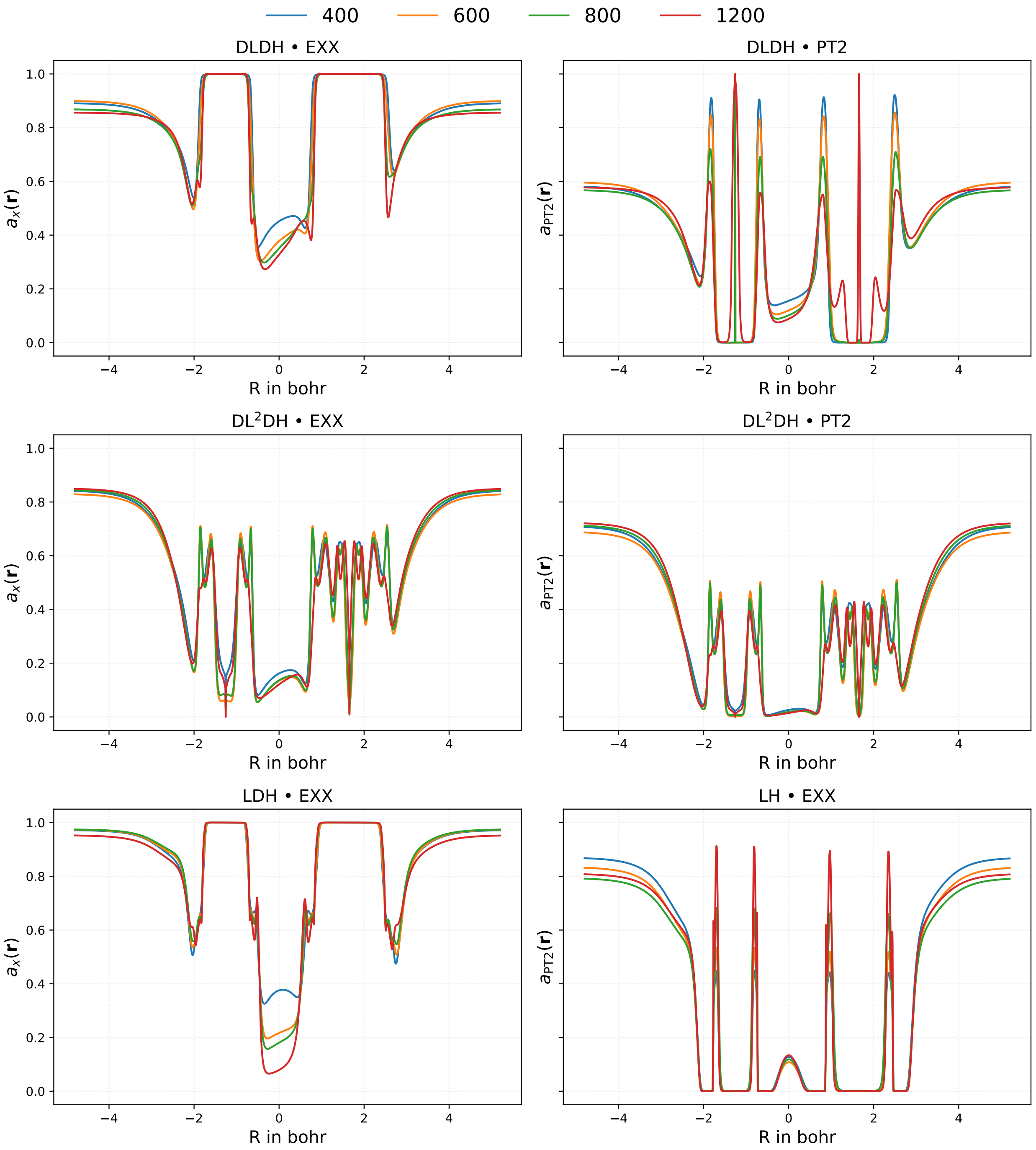

Figure S3. Evolution of LMFs for the CS molecule (with B97c) with training epoch for different types of functionals, upon training on BH76/W4-17/Slim20. Panels show the LMFs for training epochs 400, 600, 800, and 1200.

S5 Effect of the choice of training set on LMF shapes

The following figures illustrate how the LMFs for exchange and correlation depend on the choice of training data sets for the different types of functionals, after training epoch 800. For training on BH76/W4-17 (BW) (Figure **??**), the LH exchange LMFs are consistently much lower in the asymptotic region than those of the other functionals. In contrast, the LDH, DLDH, and DL^{2}DH exchange LMFs are more similar in the asymptote, but show pronounced differences in the bonding region. Notably, the LDH exchange fraction never drops below about 70% of exact exchange. For the PT2 contribution, DL^{2}DH is systematically higher than DLDH across both the asymptotic and bonding regions.

For training on BH76/W4-17/Slim16 (Figure **??**), the PT2 LMFs become significantly larger across both CS and Ar_2, for both B95c and B97c correlation, than in the other training setups. This indicates a systematically stronger local weighting of the PT2 contribution.

For training on BH76/W4-17/Slim20 (Figure **??**), a similar trend is observed for the PT2 terms with B95c correlation: DL^{2}DH remains higher than DLDH in both asymptotic and bonding regions. However, when trained with B97c correlation, the opposite holds in the bonding region, where DLDH yields the higher PT2 contribution. The exchange LMFs again show that LDH maintains a very high EXX admixture overall. Interestingly, for CS the LDH fraction now decreases to about 20% in the bond center, whereas in the BW-only training it remained much higher even in the bonding region.

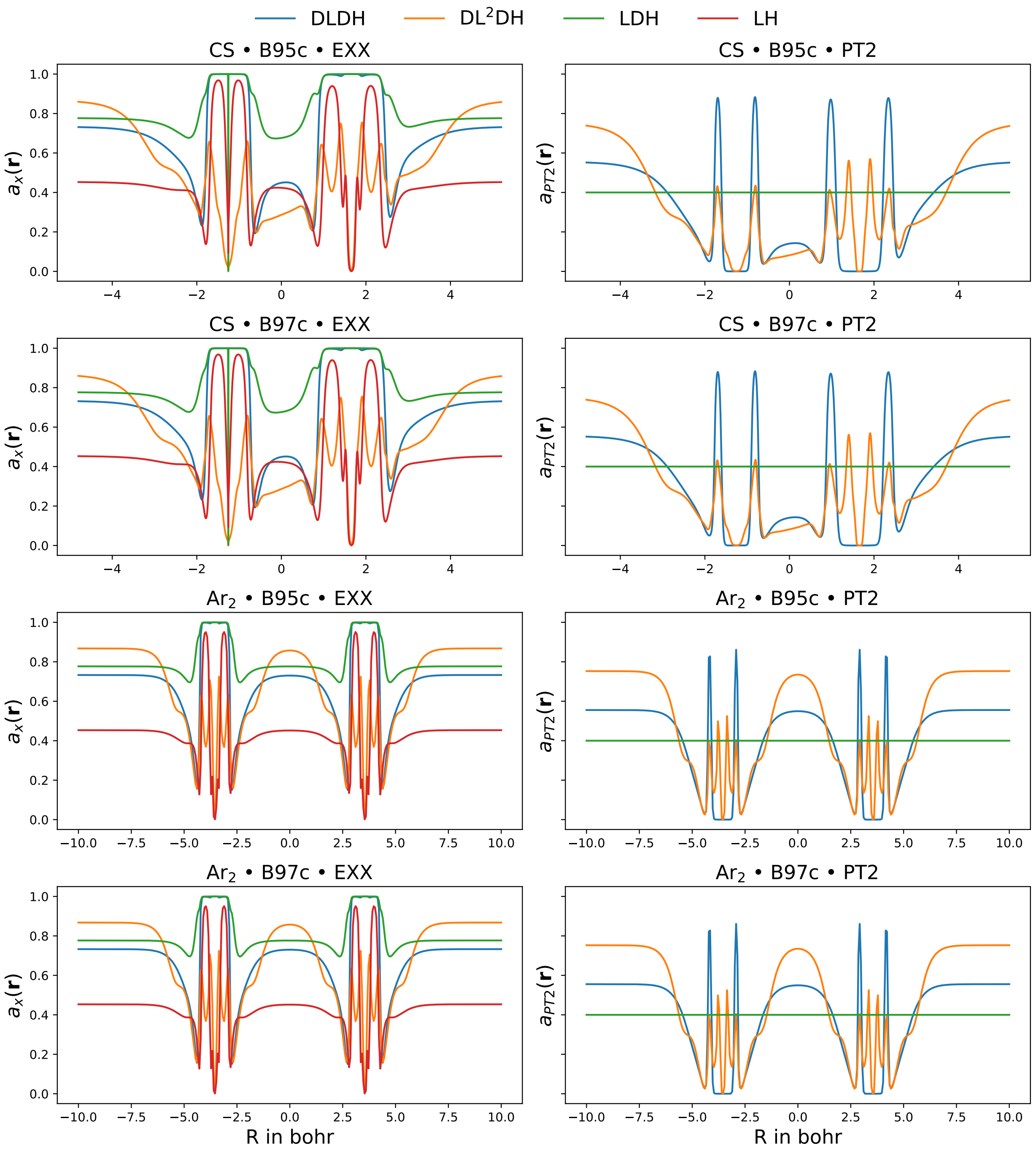

Figure S4. Plots of $a_{\mathrm{x}}(\mathbf{r})$ (left panels) and $a_{\mathrm{PT2}}(\mathbf{r})$ (right panel) for different types of functionals, trained on BH76/W4-17, for CS and Ar_2, with both B95c and B97c.

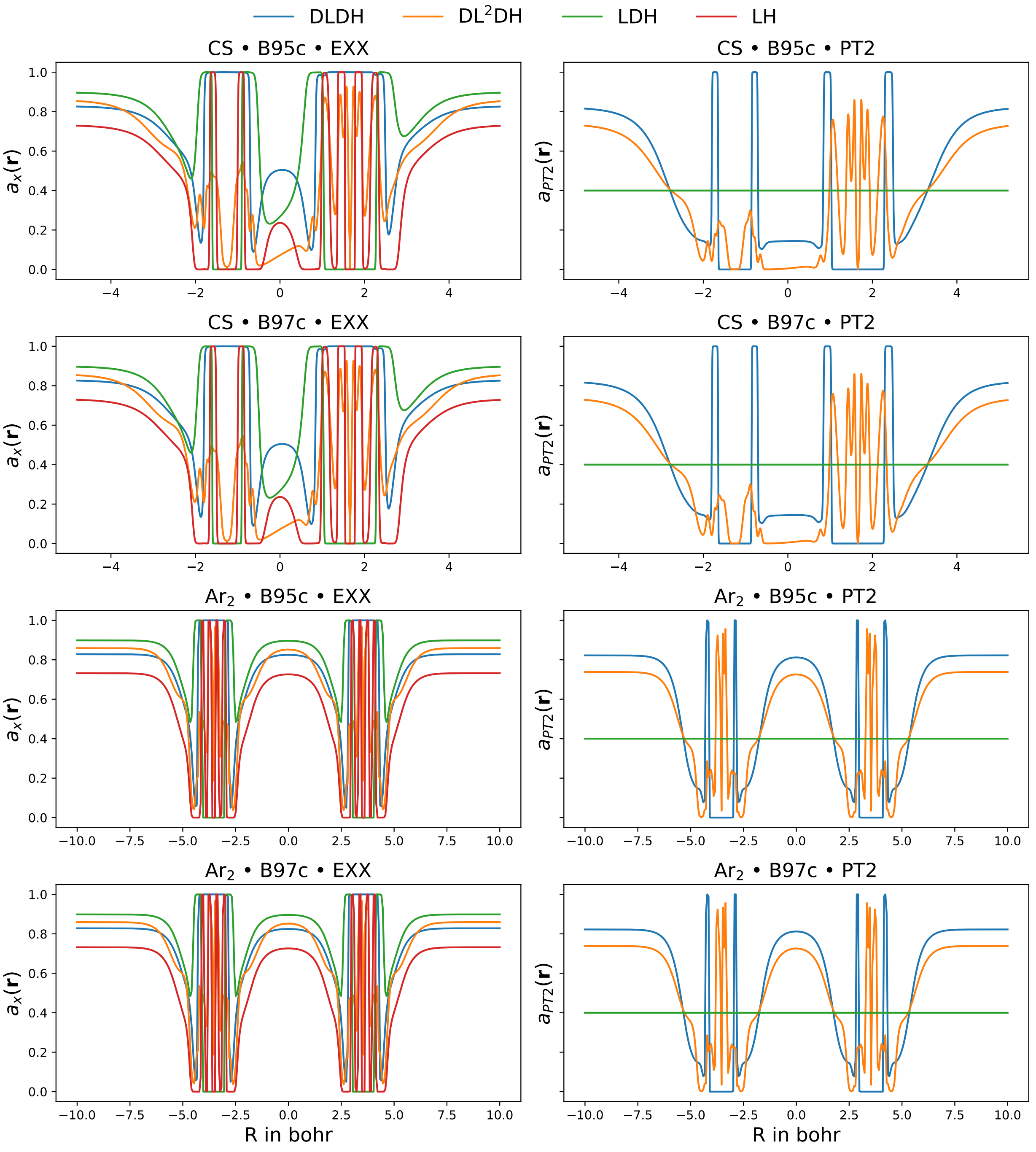

Figure S5. Plots of $a_\mathrm{x}(\mathbf{r})$ (left panels) and $a_\mathrm{PT2}(\mathbf{r})$ (right panel), trained on BH76/W4-17/Slim16, for CS and Ar_2, with both B95c and B97c.

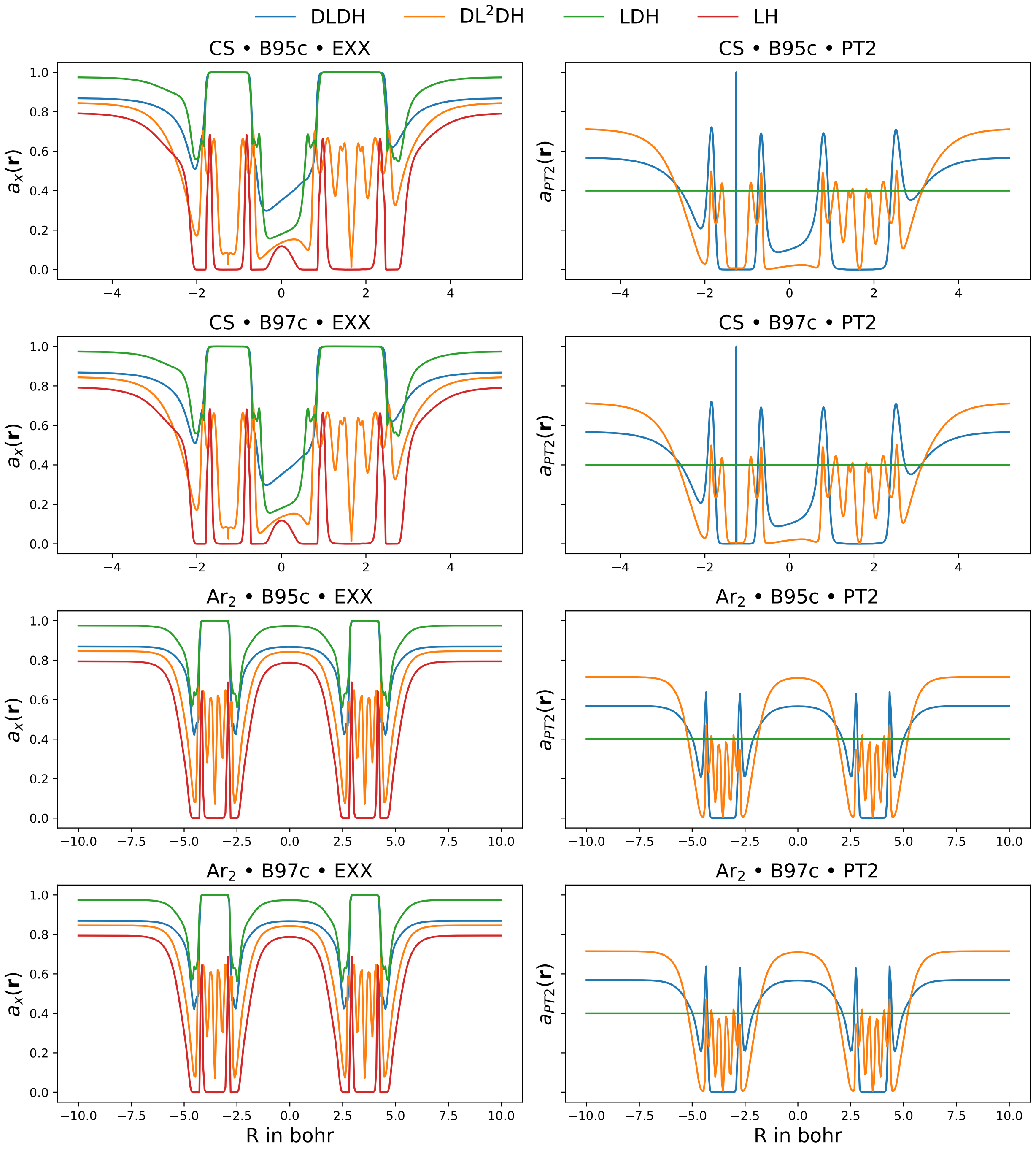

Figure S6. Plots of $a_{\mathrm{x}}(\mathbf{r})$ (left panels) and $a_{\mathrm{PT2}}(\mathbf{r})$ (right panel) for different types of functionals, trained on BH76/W4-17/Slim20, for CS and Ar_2, with both B95c and B97c.

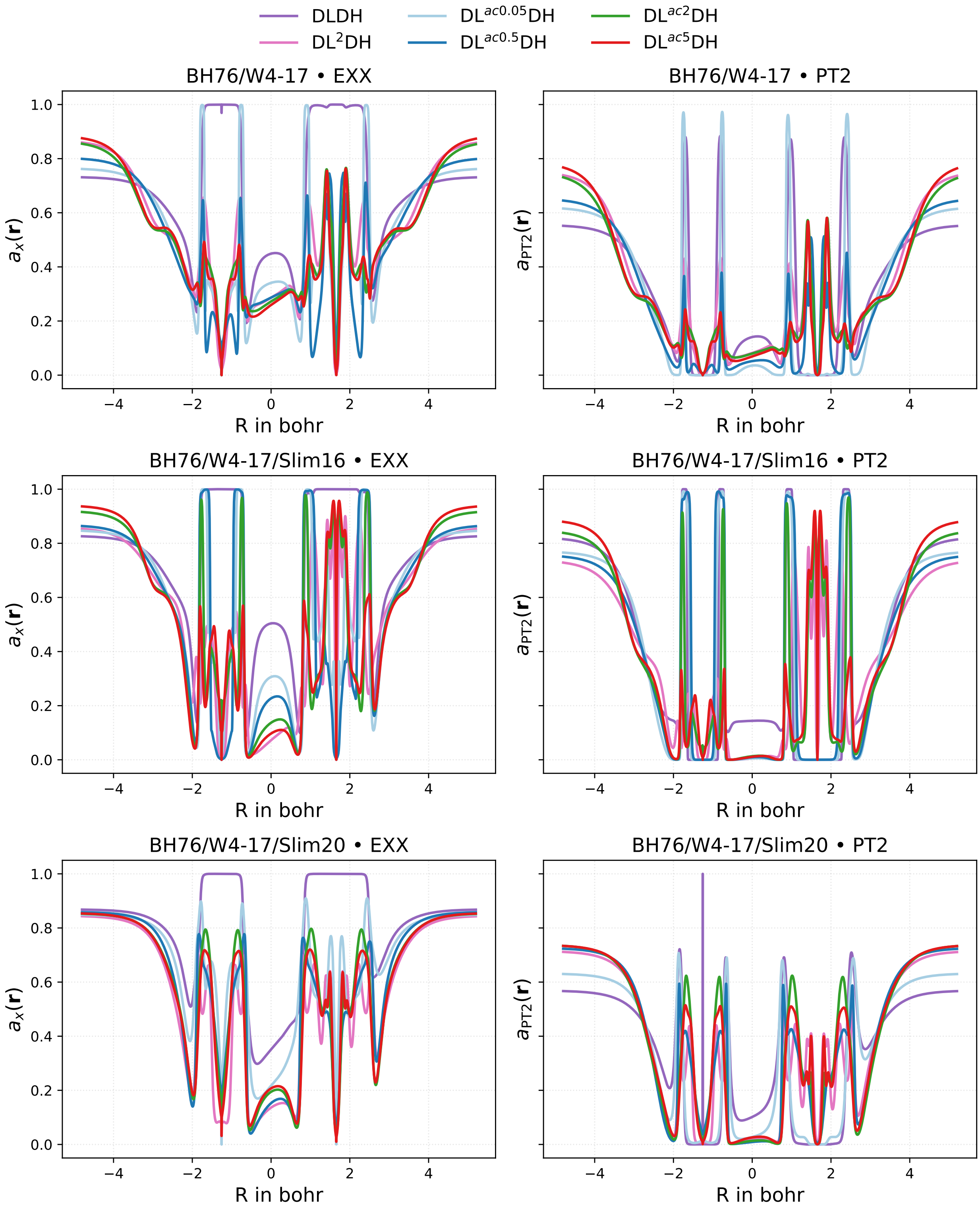

Figure S7. Plots of $a_{\mathrm{x}}(\mathbf{r})$ (left panels) and $a_{\mathrm{PT2}}(\mathbf{r})$ (right panel) for different types of functionals, first row trained on BH76/W4-17, second row trained on BH76/W4-17/Slim16 and the last row trained on BH76/W4-17/Slim20, for CS with B97c.

S6 Evaluation of WTMAD-2 values for the Slim16 and Slim20 test sets

Table S10. WTMAD-2 values in kcal/mol with DLDH, DL^{2}DH, LDH, and LH functionals on the Slim16 or Slim20 test sets for all training/evaluation setups, with B95c correlation and using dispersion corrections during training. Abbreviations: BW = BH76/W4-17, BWS16 = BH76/W4-17/Slim16, BWS20 = BH76/W4-17/Slim20. S16 = Slim16, S20 = Slim20; nD = no dispersion in the evaluation; D = dispersion in the evaluation.

Functional	Training	Evaluation	WTMAD-2 (nD)	WTMAD-2 (D)
DLDH	BW	S20	2.8116	2.6113
DLDH	BW	S16	3.4876	3.3199
DL^{2}DH	BW	S20	2.6512	2.3889
DL^{2}DH	BW	S16	3.4928	3.3712
LDH	BW	S20	2.8125	2.8304
LDH	BW	S16	3.5014	3.5347
LH	BW	S20	5.0765	3.5241
LH	BW	S16	6.5282	4.3679
DLDH	BWS16	S20	2.3618	2.4553
DL^{2}DH	BWS16	S20	1.9918	2.0086
LDH	BWS16	S20	2.5176	2.5606
LH	BWS16	S20	4.7574	4.2715
DLDH	BWS20	S16	3.6058	3.7243
DL^{2}DH	BWS20	S16	4.3347	4.3415
LDH	BWS20	S16	4.8528	4.9334
LH	BWS20	S16	5.9256	6.0023

Table S11. WTMAD-2 values in kcal/mol with DLDH, DL^{2}DH, LDH, and LH functionals on the Slim16 or Slim20 test sets for all training/evaluation setups, with B95c correlation without dispersion corrections during training. Abbreviations: BW = BH76/W4-17, BWS16 = BH76/W4-17/Slim16, BWS20 = BH76/W4-17/Slim20. S16 = Slim16, S20 = Slim20; nD = no dispersion in the evaluation; D = dispersion in the evaluation.

Functional	Training	Evaluation	WTMAD-2 (nD)	WTMAD-2 (D)
DLDH	BW	S20	2.6350	2.5481
DL^{2}DH	BW	S20	2.3829	2.1355
LDH	BW	S20	2.8076	2.7399
LH	BW	S20	5.2701	3.6170
DLDH	BW	S16	3.7642	3.7326
DL^{2}DH	BW	S16	3.3013	3.1718
LDH	BW	S16	3.5211	3.4801
LH	BW	S16	6.2922	4.2065
DLDH	BWS16	S20	2.2914	2.3368
DL^{2}DH	BWS16	S20	1.9735	2.0271
LDH	BWS16	S20	2.5233	2.5683
LH	BWS16	S20	4.5218	4.1691
DLDH	BWS20	S16	3.5361	3.6544
DL^{2}DH	BWS20	S16	4.0466	4.0537
LDH	BWS20	S16	4.7706	4.8514
LH	BWS20	S16	6.0065	6.1376

Table S12. WTMAD-2 values in kcal/mol with DLDH, DL^2DH, $DL^{ac}DH$ (approximately coupled), LDH, and LH functionals on the Slim16 or Slim20 test sets for all training/evaluation setups, with B97c correlation and no dispersion corrections during training. Abbreviations: BW = BH76/W4-17, S16BW = Slim16/BW, S20BW = Slim20/BW. S16 = Slim16, S20 = Slim20; nD = no dispersion in the evaluation; D = dispersion in the evaluation.

Functional	Training	Evaluation	WTMAD-2 (nD)	WTMAD-2 (D)
DLDH	BW	S20	2.6881	2.5752
DL^2DH	BW	S20	3.0470	2.6779
$DL^{ac0.05}DH$	BW	S20	2.6742	2.4520
$DL^{ac0.5}DH$	BW	S20	2.7637	2.5047
$DL^{ac2}DH$	BW	S20	3.3831	2.8357
$DL^{ac5}DH$	BW	S20	3.2876	2.6613
LDH	BW	S20	3.1419	3.1120
LH	BW	S20	6.5835	5.0480
DLDH	BW	S16	3.3206	3.2169
DL^2DH	BW	S16	3.7946	3.4556
$DL^{ac0.05}DH$	BW	S16	3.6317	3.3501
$DL^{ac0.5}DH$	BW	S16	3.6893	3.3995
$DL^{ac2}DH$	BW	S16	4.2568	3.6227
$DL^{ac5}DH$	BW	S16	4.1715	3.4693
LDH	BW	S16	3.4218	3.5149
LH	BW	S16	7.9998	5.9412
DLDH	S16BW	S20	2.1608	2.1588
DL^2DH	S16BW	S20	1.9784	2.0104
$DL^{ac0.05}DH$	S16BW	S20	1.9790	1.9806
$DL^{ac0.5}DH$	S16BW	S20	1.9576	1.9492
$DL^{ac2}DH$	S16BW	S20	2.0884	2.0602
$DL^{ac5}DH$	S16BW	S20	2.1290	2.1245
LDH	S16BW	S20	2.8988	2.9197
LH	S16BW	S20	4.7811	4.3210
DLDH	S20BW	S16	3.1835	3.2125
DL^2DH	S20BW	S16	3.7153	3.7251
$DL^{ac0.05}DH$	S20BW	S16	3.5117	3.5164
$DL^{ac0.5}DH$	S20BW	S16	3.7724	3.7489
$DL^{ac2}DH$	S20BW	S16	3.3292	3.3311
$DL^{ac5}DH$	S20BW	S16	3.3000	3.2990
LDH	S20BW	S16	3.8814	3.9561
LH	S20BW	S16	5.7239	5.4486

S7 Validation on the MB08-165 set

Table S13. Mean absolute errors (MAE, kcal mol^{-1}) for the DLDH, DL^2DH, LDH, and LH with B97c evaluated on the MB08-165[68] set. Training sets are denoted as BW (BH76+W4-17), S16 (BH76+W4-17+Slim16), and S20 (BH76+W4-17+Slim20).

Functional	Training set	MAE [kcal/mol]
DLDH	BW	2.11
DLDH	S16	2.81
DLDH	S20	3.02
DL^2DH	BW	3.80
DL^2DH	S16	2.55
DL^2DH	S20	3.25
LDH	BW	4.71
LDH	S16	3.33
LDH	S20	5.17
LH	BW	7.24
LH	S16	9.81
LH	S20	8.75
CCSD/cc-pVQZ[a]	—	5.64
CCSD(T)/cc-pVQZ[a]	—	2.61
CCSD(T)/cc-pVTZ[a]	—	5.45
B2-PLYP-D[a]	—	4.09
M06-2X[a]	—	4.82

[a]Reference values were taken from Ref. S 68.

S8 Validation on the BH76RC set

Table S14. Mean absolute errors (MAE, kcal mol^{-1}) for the DLDH, DL^{2}DH, LDH, and LH models with B97c evaluated on the BH76RC[?] reaction-energy benchmark. Training sets are denoted as BW (BH76+W4-17), S16 (BH76+W4-17+Slim16), and S20 (BH76+W4-17+Slim20).

Functional	Training set	MAE [kcal/mol]
DLDH	BW	1.17
DLDH	S16	0.92
DLDH	S20	0.97
DL^{2}DH	BW	1.54
DL^{2}DH	S16	0.89
DL^{2}DH	S20	0.75
LDH	BW	1.97
LDH	S16	1.48
LDH	S20	1.41
LH	BW	2.35
LH	S16	1.79
LH	S20	1.57
DH23[a]	—	0.94
ωB97M(2)[a]	—	0.81
XYG8[a]	—	0.98
revDSD[a]	—	0.79
xDSD[a]	—	0.72

[a]Reference values were taken from Ref. S **?** .

S9 Argon - benzene dissociation curves

The B95c-based functionals exhibit noticeable non-smoothness in the argon–benzene dissociation curves even with the (99,590) grid, while for B97c-based functionals such irregularities appear only for the coarser PySCF grid level 1. Similar grid-related effects for this system were also observed in the ωB97M-V study.[66]

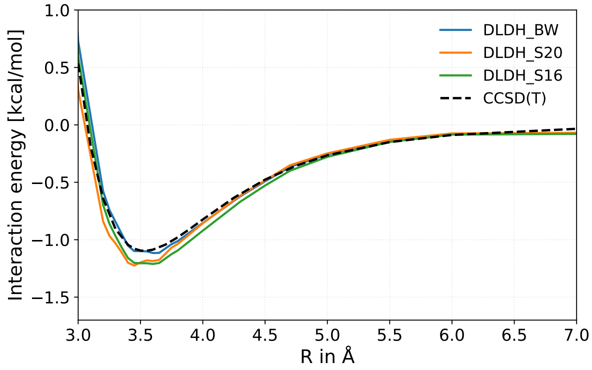

(a) DLDH

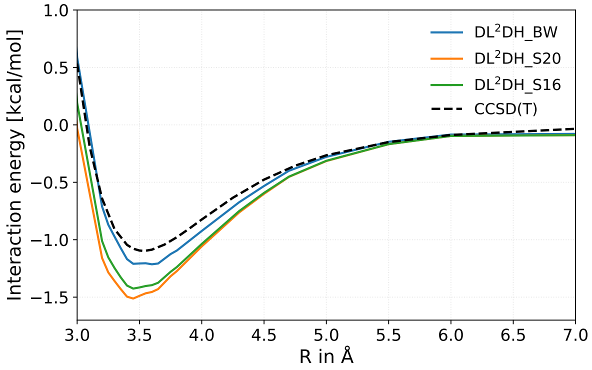

(b) DL^{2}DH

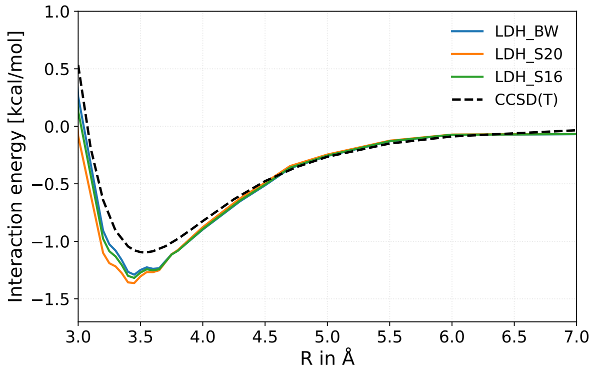

(c) LDH

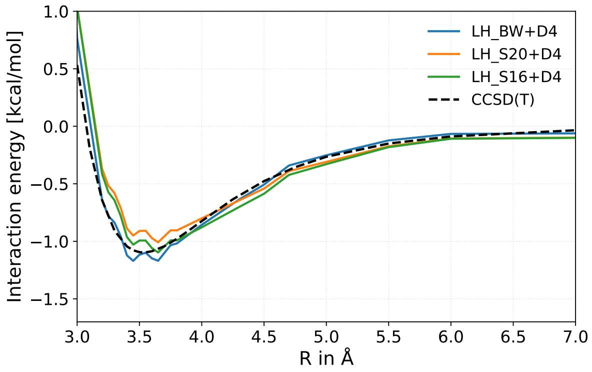

(d) LH-D4

Figure S8. Dissociation curves of the argon–benzene complex obtained with B95c-based functionals, using PySCF with grid (99, 590), compared to the CCSD(T) reference curve.

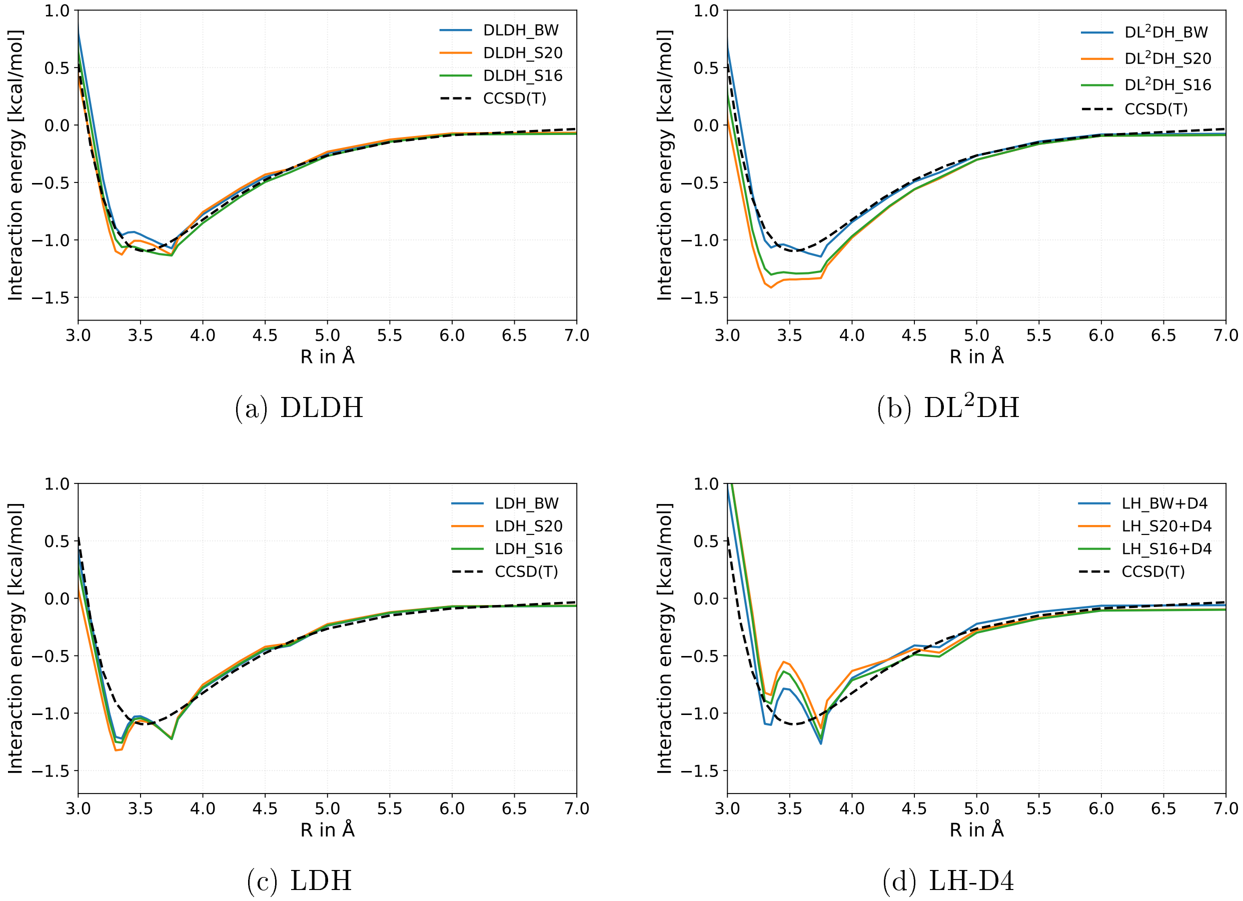

Figure S9. Dissociation curves of the argon–benzene complex obtained with B95c-based functionals, using PySCF with grid level 1, compared to the CCSD(T) reference curve.

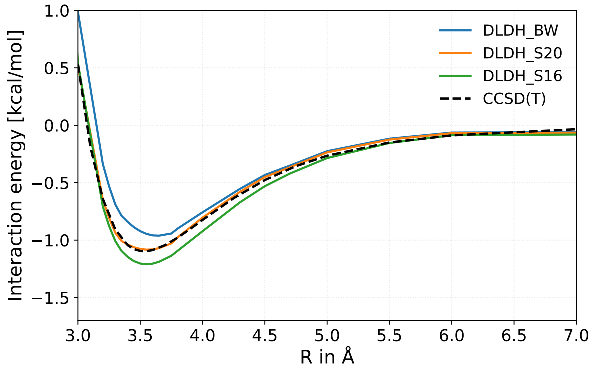

(a) DLDH

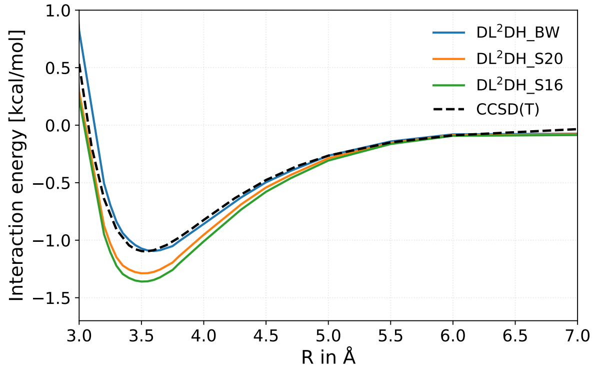

(b) DL^{2}DH

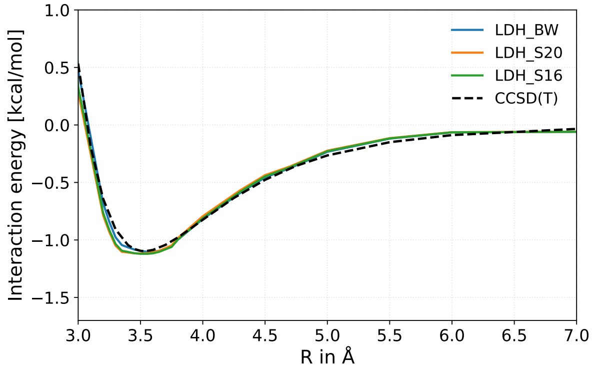

(c) LDH

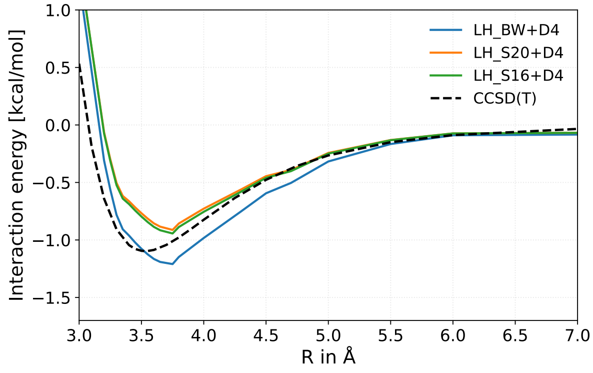

(d) LH-D4

Figure S10. Dissociation curves of the argon–benzene complex obtained with B97c-based functionals, using PySCF with grid level 1, compared to the CCSD(T) reference curve.

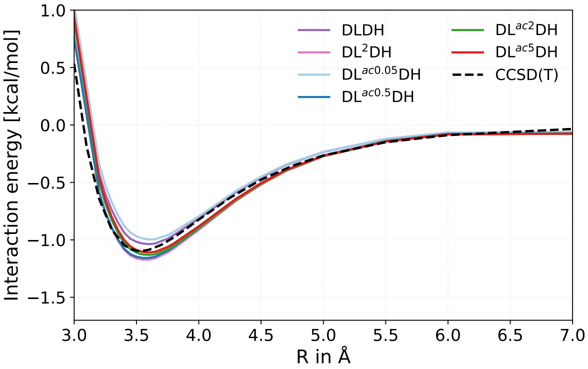

(a) Trained on BH76/W4-17

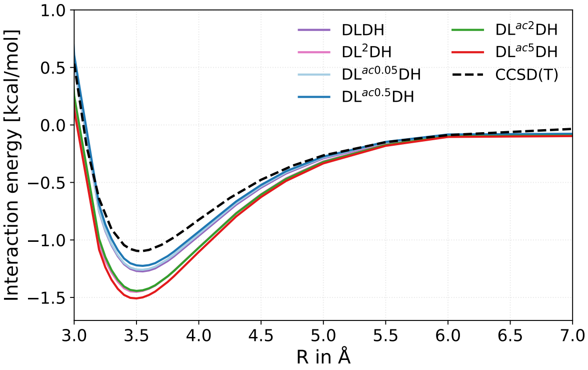

(b) Trained on BH76/W4-17/Slim16

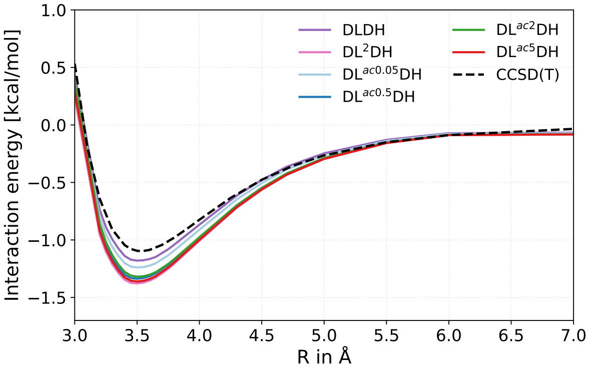

(c) Trained on BH76/W4-17/Slim20

Figure S11. Dissociation curves of the argon–benzene complex obtained with B97c-based functionals including the "approximetly coupled' DLDH functionals as well as the DLDH and DL^{2}DH, using PySCF with grid (99, 590), compared to the CCSD(T) reference curve.
